# Highly Basic Clusters in the Herpes Simplex Virus 1 Nuclear Egress Complex Drive Membrane Budding by Inducing Lipid Ordering

**DOI:** 10.1128/mBio.01548-21

**Published:** 2021-08-24

**Authors:** Michael K. Thorsen, Alex Lai, Michelle W. Lee, David P. Hoogerheide, Gerard C. L. Wong, Jack H. Freed, Ekaterina E. Heldwein

**Affiliations:** a Department of Molecular Biology and Microbiology, Graduate Program in Cellular, Molecular and Developmental Biology, Tufts University School of Medicine, Boston, Massachusetts, USA; b Department of Chemistry and Chemical Biology and National Biomedical Center for Advanced Electron Spin Resonance Technology, Cornell University, Ithaca, New York, USA; c Department of Bioengineering, Department of Chemistry and Biochemistry, California NanoSystems Institute, University of California, Los Angeles, Los Angeles, California, USA; d Center for Neutron Research, National Institute of Standards and Technology, Gaithersburg, Maryland, USA; Princeton University

**Keywords:** HSV-1, nuclear egress, membrane interactions, membrane budding, membrane curvature, electron spin resonance, neutron reflectometry, small-angle X-ray scattering, charge clusters, electrostatics, herpes simplex virus, herpesviruses, membrane deformation, complex, phosphorylation

## Abstract

During replication of herpesviruses, capsids escape from the nucleus into the cytoplasm by budding at the inner nuclear membrane. This unusual process is mediated by the viral nuclear egress complex (NEC) that deforms the membrane around the capsid by oligomerizing into a hexagonal, membrane-bound scaffold. Here, we found that highly basic membrane-proximal regions (MPRs) of the NEC alter lipid order by inserting into the lipid headgroups and promote negative Gaussian curvature. We also find that the electrostatic interactions between the MPRs and the membranes are essential for membrane deformation. One of the MPRs is phosphorylated by a viral kinase during infection, and the corresponding phosphomimicking mutations block capsid nuclear egress. We show that the same phosphomimicking mutations disrupt the NEC-membrane interactions and inhibit NEC-mediated budding *in vitro*, providing a biophysical explanation for the *in vivo* phenomenon. Our data suggest that the NEC generates negative membrane curvature by both lipid ordering and protein scaffolding and that phosphorylation acts as an off switch that inhibits the membrane-budding activity of the NEC to prevent capsid-less budding.

## INTRODUCTION

To overcome the barriers presented by compartmentalization in eukaryotic cells, viruses must manipulate cellular membranes. One of the more unusual mechanisms of membrane remodeling is found in herpesviruses: large double-stranded-DNA viruses that infect nearly all vertebrates and some invertebrates for life (1) and, in humans, can cause symptoms ranging from painful skin lesions to blindness and life-threatening conditions in people with weak or immature immune systems (2). After viral genomes are replicated and packaged, herpesviral capsids traverse several host membrane barriers to complete their assembly and exit the cell as infectious virions (reviewed in references 3–6). The critical, conserved first step in this process is nuclear egress, during which newly formed capsids translocate from the nucleus into the cytoplasm. Many viruses that replicate their genomes within the nucleus exit this double-membraned organelle through nuclear pores, such as HIV (uncoated genome), influenza (protein-coated genome), and polyomaviruses (complete viral particle) (7, 8). However, the ∼50-nm opening of the nuclear pore is too small to accommodate the ∼125-nm herpesviral capsids. Instead, herpesviruses use a different, noncanonical nuclear export mechanism where capsids acquire envelopes by budding at the inner nuclear membrane (INM) and pinching off into the perinuclear space. These perinuclear enveloped virions then fuse with the outer nuclear membrane (ONM), releasing the capsids into the cytoplasm (reviewed in references 3, 5, 6, 9, 10).

Capsid budding at the INM requires the generation of negative membrane curvature by the viral nuclear egress complex (NEC), a heterodimer of two conserved viral proteins: UL31, a soluble nuclear phosphoprotein, and UL34, which contains a single C-terminal transmembrane (TM) helix that anchors the NEC in the INM (reviewed in reference 5). Both UL31 and UL34 are essential for nuclear egress, and in the absence of either protein, capsids accumulate in the nucleus and the production of infectious virions is significantly impaired (11–20). Using *in vitro* model systems and cryogenic electron microscopy and tomography (cryo-EM/ET), we previously discovered that the NEC from a prototypical herpes simplex virus 1 (HSV-1) vesiculates synthetic lipid bilayers *in vitro* in the absence of any other factors or ATP (21), which was later confirmed with the NEC homolog from a closely related pseudorabies virus (PRV) (22). Likewise, overexpression of PRV or Kaposi’s sarcoma-associated herpesvirus (KSHV) NEC in uninfected cells caused formation of capsidless vesicles in the perinuclear space (23, 24). Furthermore, cryo-EM studies showed that the NEC oligomerizes into hexagonal scaffold-like coats on the inner surface of budded vesicles formed *in vitro* (21), in cells overexpressing PRV NEC (25), and in perinuclear enveloped vesicles purified from HSV-infected cells (26). NEC oligomerization is necessary for budding, because mutations intended to disrupt oligomeric interfaces reduce budding both *in vivo* and *in vitro* (21, 27–29). Collectively, these findings established the NEC as a robust membrane-budding machine that forms hexagonal scaffolds (reviewed in reference 5).

Although NEC oligomerization is required for budding, NEC-membrane interactions may also have a mechanistic role in its budding mechanism. The TM helix of UL34 seemingly functions only to anchor the NEC to the INM (30) because it is dispensable for budding *in vitro* (21) and can be replaced with a heterologous TM *in vivo* (30). However, both UL31 and UL34 homologs have highly basic membrane-proximal regions (MPRs), and *in vitro* budding by HSV-1 or PRV NEC requires acidic lipids (21, 22), which implicates electrostatic interactions. Moreover, MPRs recruit the recombinant soluble HSV-1 NEC (which lacks the transmembrane anchor yet maintains robust budding activity) to acidic membranes *in vitro* (21). It is unclear, however, how the MPRs interact with membranes or how these interactions lead to the formation of the negative membrane curvature during budding. Additionally, the HSV-1 UL31 MPR is phosphorylated during infection (31) by the viral kinase US3 (32) that targets six serines (33). The role of UL31 phosphorylation in nuclear egress is unclear, but phosphomimicking serine-to-glutamate mutations of these six serines inhibits nuclear egress and HSV-1 replication (33), suggesting that phosphorylation inhibits nuclear egress, by an unknown mechanism, presumably to prevent unproductive budding prior to the arrival of the capsid (reviewed in reference 5). Thus, the MPRs may have both mechanistic and regulatory roles in NEC-mediated membrane budding. It is unknown how the MPR-membrane interactions generate negative membrane curvature necessary for budding.

In addition to generating membrane buds, the NEC can also sever the necks of the budded vesicles at least *in vitro* (21) and, potentially, in some infected cell types (34), even though in other cell types the cellular ESCRT-III machinery is recruited for scission (35). Thus, another important unanswered question is how the NEC can generate both the membrane curvature necessary for the formation of the bud and a very different nanoscopic curvature required for scission to complete the budding process.

Here, by employing mutagenesis and several biophysical methods, we show that highly basic MPRs of the NEC are required for budding, can induce ordering within the headgroup and acyl chain regions of lipids in synthetic membranes, and can promote negative Gaussian curvature, which is the distinct type of curvature required for membrane scission. We propose that the NEC generates negative membrane curvature by a mechanism that combines lipid ordering and protein scaffolding. We also show that membrane remodeling by the NEC requires electrostatic interactions between the basic clusters within the MPRs and the acidic membranes. Further, we show evidence that the virus controls the membrane-budding activity of the NEC by manipulating its membrane interactions through phosphorylation, which would reduce the electrostatic interactions. Specifically, we demonstrate that the phosphomimicking mutations of serines adjacent to the basic clusters inhibit NEC-mediated budding *in vitro*, which explains how these mutations can also block capsid nuclear egress. HSV-1 may use phosphorylation to inhibit unproductive budding in the absence of the capsid by reducing the membrane-budding activity of the NEC.

## RESULTS

### The MPRs are required for efficient NEC-mediated membrane budding *in vitro*.

**FIG 1:**
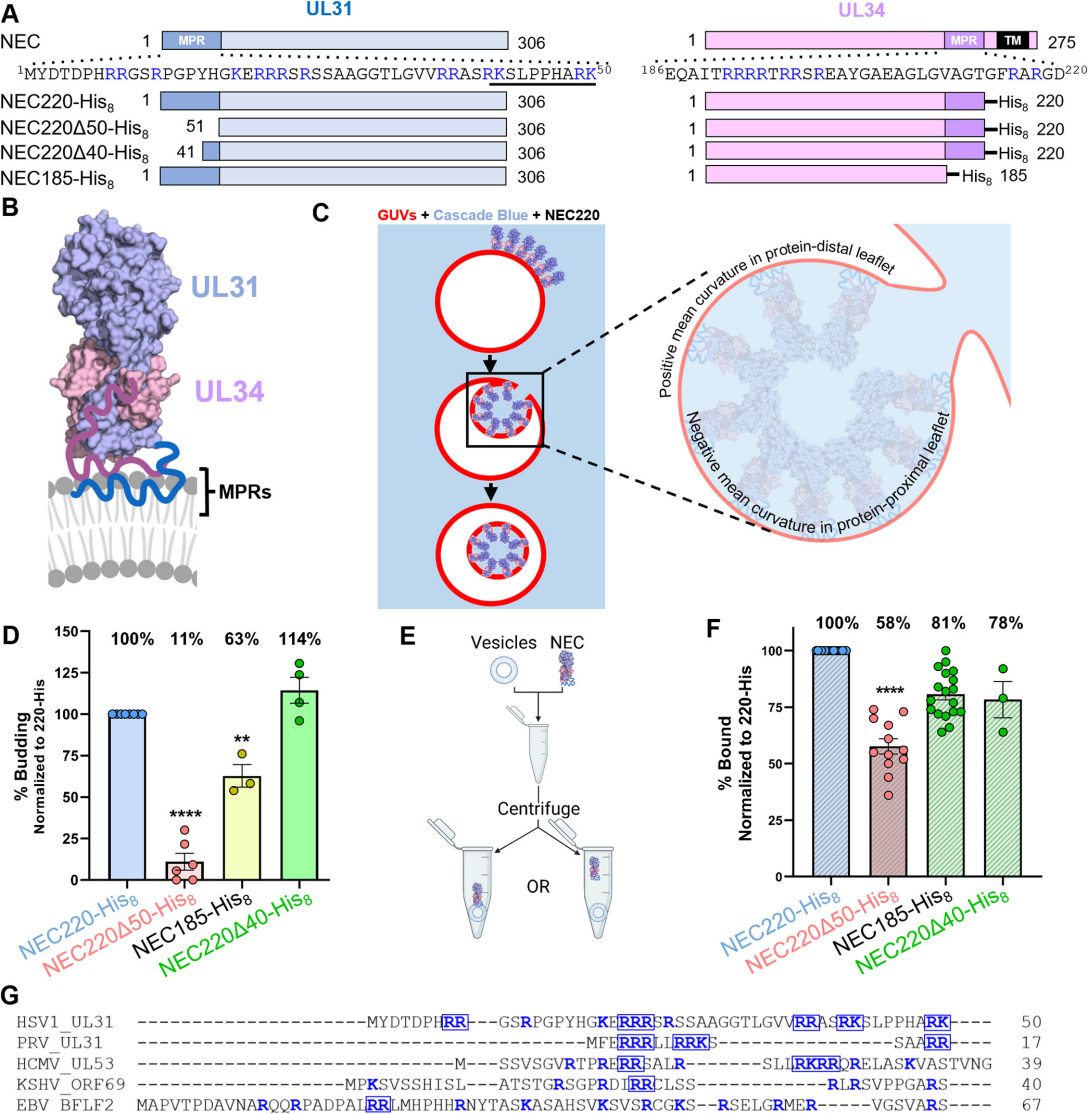
NEC MPRs are necessary for membrane vesiculation. (A) NEC construct map. Sequence of UL31 MPR residues 1 to 50 and UL34 MPR residues 186 to 220 shown at top. Basic residues are in blue, UL31 mini-MPR is underlined. (B) Crystal structure of HSV-1 NEC. MPRs missing from the structure are shown schematically in blue (UL31) and purple (UL34). UL34 TM is not included in the schematic. Image generated using BioRender (BioRender.com). (C) *In vitro* budding assay. Red-labeled GUVs are incubated with NEC in buffer containing Cascade Blue, a membrane-impermeant dye. Upon a budding event, intraluminal vesicles will form, allowing blue dye into the red-labeled GUV. Inset shows a budding vesicle depicting two types of mean curvature. Inset made with BioRender.com. (D) Vesicles contain Ni-chelating lipids to tether His_8_-tagged NEC to membranes. Percent budding was determined by counting the number of ILVs after addition of NEC and then normalized to NEC220-His_8_ amounts. Background levels of ILVs in the absence of NEC were subtracted from all values before normalization. Significance to 220Δ40-His_8_ was calculated using an unpaired Student's *t* test with Welch’s correction (**, *P* < 0.005; ***, *P* < 0.0005). In all plots, error bars represent the standard error of the mean (68% confidence interval of the mean) for at least three individual experiments. (E) *In vitro* cosedimentation assay. Vesicles are incubated with NEC and then spun down in a centrifuge. Samples of the supernatant and pellet are run on an SDS-PAGE gel to determine the amount of NEC that pelleted with vesicles. Image made using Biorender.com. (F) Percent bound was determined by quantification of SDS-PAGE gels of NEC with or without vesicles. Each bar represents the amount of protein pelleted. Significance to 220Δ40-His_8_ was calculated using an unpaired Student's *t* test with Welch’s correction (****, *P* < 0.0001). (G) Multiple-sequence alignment of HSV-1, PRV, HCMV, KSHV, and EBV UL31 MPRs performed with Clustal omega (40). Basic residues are shown in blue. Charge clusters are boxed.

HSV-1 UL31 is a soluble 306-amino-acid protein, and HSV-1 UL34 is a 275-amino-acid protein with a single C-terminal TM helix (Fig. 1A). The highly basic MPRs encompass residues 1 to 50 of UL31 and 186 to 220 of UL34, which are absent from the crystal structures of the NEC cores and are located at the membrane-proximal end of the NEC (Fig. 1B) (27). Previously, using an *in vitro* budding assay with giant unilamellar vesicles (GUV) (Fig. 1C), we showed that the NEC construct containing full-length UL31 and residues 1 to 220 of UL34 (NEC220) (Fig. 1A) mediated robust membrane budding *in vitro* (21). We also showed that the MPRs were necessary to recruit the NEC220 to synthetic membranes (21) but did not investigate the potential role of the MPRs in the budding process beyond membrane recruitment, partly because the soluble NEC220 must be recruited to the membranes from bulk solvent, making it difficult to uncouple NEC-membrane interactions necessary for budding from those necessary for membrane recruitment. To overcome this challenge, we utilized an NEC220 variant containing a C-terminal His_8_ tag in UL34 (21). When used in conjunction with Ni-chelating lipids in the liposomes (36), polyhistidine tags efficiently tether proteins to membranes and are often used in place of TM anchors. The resulting NEC220-His_8_ construct had the same budding efficiency as the untagged NEC220 (21).

By deleting the MPRs individually from the NEC220-His_8_ parent construct, we found that while both MPRs were required for efficient membrane budding *in vitro*, the UL31 MPR was more important, because its deletion (NEC220Δ50-His_8_) reduced membrane budding to a very low level (11% ± 5% standard error of the mean relative to NEC220-His_8_), whereas the deletion of the UL34 MPR (NEC185-His_8_) maintained budding at a moderate level (63% ± 7%) (Fig. 1D). To assess the effect of MPR deletions on membrane recruitment, we used a cosedimentation assay described previously (Fig. 1E) (21) with synthetic membranes lacking nickel-nitrilotriacetic acid (Ni-NTA)-conjugated lipids but containing 40% negatively charged lipids, which are required for membrane recruitment of the soluble NEC220 (21). NEC220Δ50-His_8_ exhibited a large decrease in membrane association (58% ± 3%) relative to NEC220-His_8_, whereas the membrane association of NEC185-His_8_ was only moderately reduced (78% ± 8%) (Fig. 1F), which suggested that the UL31 MPR is more important for both membrane recruitment and budding activity than the UL34 MPR.

To narrow down residues within the UL31 MPR (Fig. 1A) responsible for membrane interactions, we tested the truncation mutant NEC220Δ40-His_8_ that lacks residues 1 to 40 of the UL31 MPR (Fig. 1A). Previously, we showed that these residues were dispensable for the membrane recruitment of soluble NEC220 (21). Here, we found that these residues were also dispensable for budding (Fig. 1D and F). Therefore, residues 41 to 50 can substitute for the full-length UL31 MPR during budding *in vitro*, and we refer to them as the “mini-MPR.”

### Basic clusters within the UL31 mini-MPR are essential for efficient budding.

Due to its size, the mini-MPR of UL31 (^41^RKSLPPHARK^50^) provides an opportunity to dissect sequence requirements for NEC-membrane interactions and budding in a simplified system. Therefore, mutations were introduced into the NEC220Δ40-His_8_ parent construct. We first explored the role of the basic residues because electrostatic interactions between basic residues and acidic lipids commonly serve to recruit cytoplasmic proteins to membranes (37), and the MPRs of UL31 and UL34 homologs are rich in basic residues, 14 in HSV-1 UL31 (28%) and 9 in HSV-1 UL34 (31%) (Fig. 1A and G). Additionally, membrane binding by soluble HSV-1 NEC requires acidic lipids and is inhibited by high NaCl concentrations (21), which further implicates electrostatic forces in NEC-membrane interactions.

**FIG 2:**
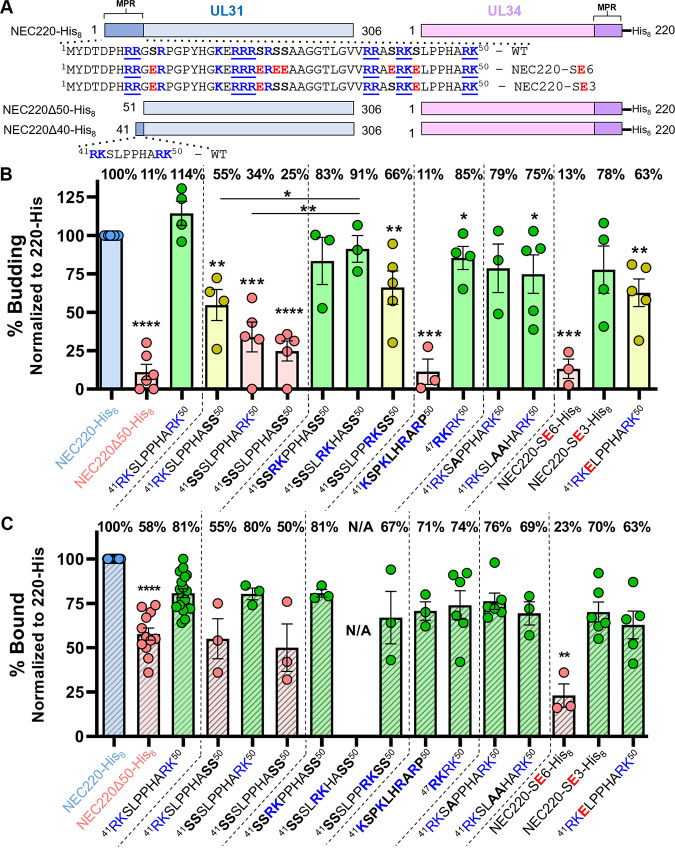
Altering the location of basic residues in the UL31 mini-MPR and introducing phosphomimicking mutations influence budding. (A) NEC construct map. Basic residues are boldfaced and blue, clusters are underlined. Phosphorylatable serines are boldfaced. Serine-to-glutamate phosphomimicking mutants are boldfaced and red. (B) *In vitro* budding assay. Mutated residues are shown in boldface. Percent budding was determined by counting the number of ILVs after addition of NEC and then normalized to NEC220-His_8_ amounts. Background levels of ILVs in the absence of NEC were subtracted from all values before normalization. Data for NEC220-His_8_, NEC220Δ50-His_8_, and NEC220Δ40-His_8_ (shown here as ^41^RKSLPPHARK^50^) are copied from Fig. 1D. Significance to 220Δ40-His_8_ or 220Δ40-P45R/P46K-His_8_ was calculated using an unpaired Student's *t* test with Welch’s correction (*, *P* < 0.05; **, *P* < 0.005; ***, *P* < 0.0005; ****, *P* < 0.0001). In both plots, error bars represent the standard error of the mean (68% confidence interval of the mean) for at least three individual experiments. Coloring scheme based on significance: 0 to 49% is poor budding (red), 50 to 74% is moderate (yellow), and 75 to 100% is efficient (green). (C) *In vitro* cosedimentation assay. Percent bound fraction was determined by quantification of SDS-PAGE gels of NEC with or without vesicles. Each bar represents the amount of protein pelleted. Binding values are to the right of the graph. Data for NEC220-His_8_, NEC220Δ50-His_8_, and NEC220Δ40-His_8_ (shown here as ^41^RKSLPPHARK^50^) are copied from Fig. 1F. Data are normalized to NEC220-His_8_. Significance relative to NEC220Δ40-His_8_ was calculated using an unpaired Student's *t* test with Welch’s correction (**, *P* < 0.007; ****, *P* < 0.0001).

The mini-MPR of UL31 has four basic residues arranged into two dibasic motifs, R41/K42 and R49/K50 (Fig. 2A), so we mutated them individually or together to serines to maintain the polar character of the side chains (Fig. 2B). Both dibasic motifs were required for efficient budding, because the mutant containing only the first dibasic motif (NEC220Δ40-R49S/K50S-His_8_) maintained moderate budding efficiency (55% ± 10%), whereas the mutants containing only the second dibasic motif (NEC220Δ40-R41S/K42S-His_8_) or no dibasic motifs (NEC220Δ40-R41S/K42S/R49S/K50S-His_8_) budded membranes inefficiently at 34% ± 10% and 25% ± 6%, respectively (Fig. 2B). To probe the importance of charge distribution within the mini-MPR, we relocated the single dibasic motif, generating mutants NEC220Δ40-S43R/L44K-His_8_, NEC220Δ40-P45R/P46K-His_8_, and NEC220Δ40-H47R/A48K-His_8_. All three mutants mediated efficient budding, but NEC220Δ40-P45R/P46K-His_8_ budded membranes more efficiently than mutants with a single dibasic motif at the N terminus or C terminus (Fig. 2B), suggesting that the location of the basic cluster can influence the budding efficiency.

In the case of membrane association, a single dibasic motif sufficed for efficient membrane association (80% ± 3%, 81% ± 2%, and 67% ± 15%) unless it was located at the N terminus, in which case membrane association was poor (55% ± 11%) (Fig. 2C). Membrane association of NEC220Δ40-P45R/P46K-His_8_ could not be assessed because protein aggregated when incubated at room temperature for >15 min (Fig. 2C). Distinct effects of dibasic motif mutations on budding versus membrane association suggest that the requirements for efficient budding versus membrane recruitment differ.

To probe the importance of charge clustering, we generated the scrambled mutant NEC220Δ40scr-His_8_ (^41^KSPKLHRARP^50^) that lacked basic clusters yet maintained the overall net +4 charge. This mutant associated efficiently with membranes (71% ± 5%) (Fig. 2C) yet mediated budding at a minimal level (11% ± 8%) (Fig. 2B), demonstrating the most pronounced difference between the requirements for budding versus membrane recruitment. Thus, whereas membrane association requires a positive net charge of at least +2, membrane budding additionally requires charge clustering.

We also investigated the role of the LPP sequence in the middle of the mini-MPR. L44 is the sole hydrophobic residue within the mini-MPR, and hydrophobic interactions can contribute to protein-membrane interactions (37), whereas the rigid di-proline motif in the middle of the mini-MPR could, in principle, adopt secondary structures important for membrane interactions. However, both the NEC220Δ40-L44A-His_8_ and the NEC220Δ40-P45A/P46A-His_8_ mutants supported efficient budding (Fig. 2B); therefore, the LPP sequence does not appear to play any role in either budding or membrane association.

To determine if 4 basic residues could replace the mini-MPR, we generated the NEC220Δ50-RKRK-His_8_ mutant. This mutant supported efficient budding (85% ± 8%) (Fig. 2B) and membrane association (74% ± 8%) (Fig. 2C). Thus, basic clusters are both necessary and sufficient for NEC-mediated budding *in vitro*. Similarly, the replacement of the UL31 MPR in PRV with 4 basic residues maintained efficient nuclear egress and replication of PRV (38).

### Phosphomimicking mutations reduced both membrane association and budding.

**FIG 3:**
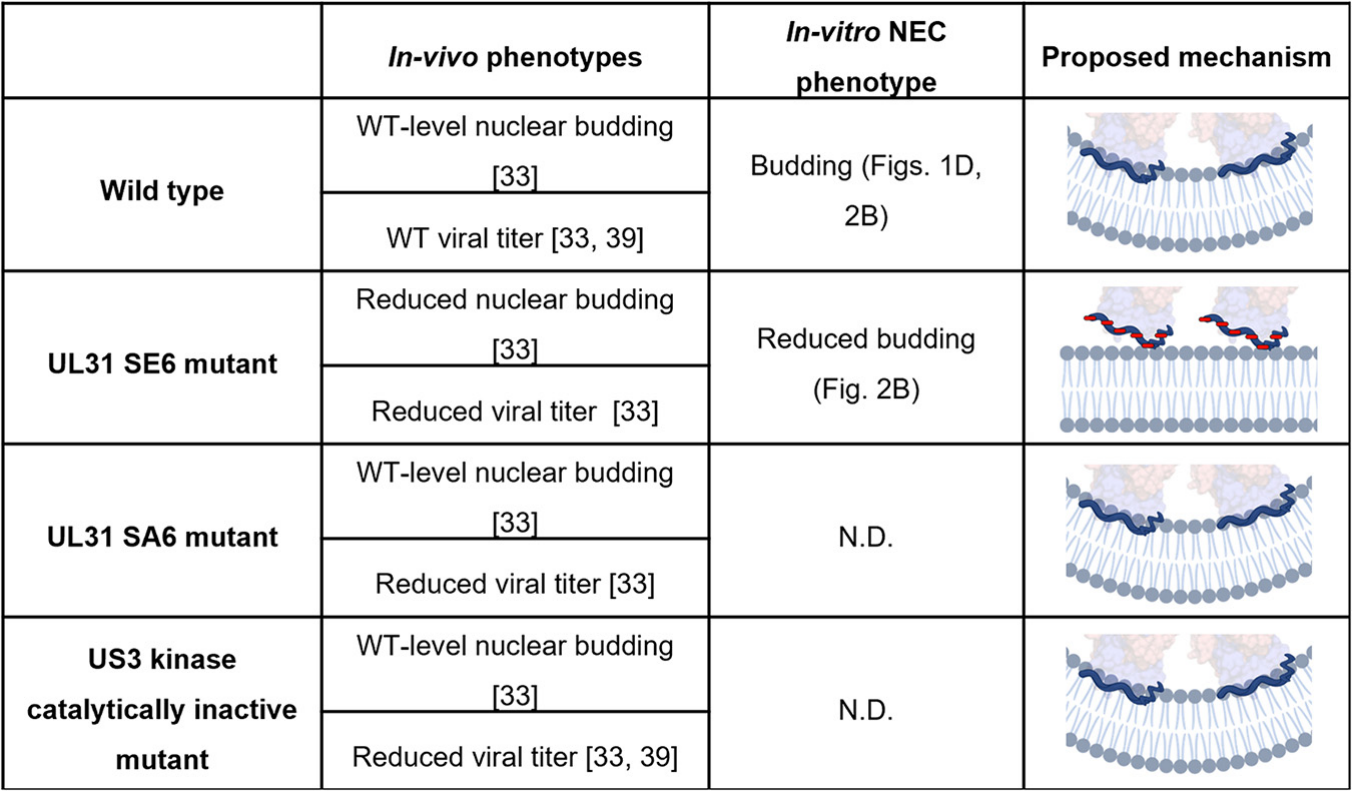
Summary of *in vivo* and *in vitro* UL31 MPR phosphorylation phenotypes. The effect of UL31 MPR phosphorylation status on nuclear budding and viral titer *in vivo* is shown side by side with the *in vitro* budding phenotypes (ND, not determined). The proposed mechanism shows induction of membrane curvature in the absence of phosphorylation due to optimal membrane interactions but no membrane curvature upon the introduction of negative charges (red rectangles) via phosphomimicking mutations that are expected to perturb membrane interactions. The UL31 MPR is shown schematically in blue; the crystal structure of the NEC is shown as a transparent surface.

HSV-1 UL31 MPR is phosphorylated during infection (31) by the viral kinase US3 (32), which targets six serines, S11, S24, S26, S27, S40, and S43 (33). The role of UL31 phosphorylation in nuclear egress has not yet been elucidated fully. Whenever UL31 cannot be phosphorylated, either due to a missing or a catalytically inactive US3 kinase (33, 39, 41, 42) or due to the substitution of these six serines for alanines, which mimics an unphosphorylated state, budded capsids accumulate in the perinuclear space and the viral titers are reduced (Fig. 3) (33). Nevertheless, phosphomimicking mutations of these six serines (serine-to-glutamate) reduce nuclear egress and HSV-1 titers (33), which suggests that phosphorylation inhibits nuclear egress by an unknown mechanism.

We have shown that positive charges in UL31 MPR are important for both the membrane association and the budding activity of the NEC. By decreasing the net positive charge of the UL31 MPR, the negative charges introduced by the phosphomimicking mutations would be expected to reduce both membrane association and the budding activity of the NEC. To test this, we generated the NEC220-SE6-His_8_ mutant, in which six serines within UL31 MPR were replaced with glutamates. Indeed, the phosphomimicking mutant had poor budding activity (13% ± 6%) (Fig. 2B) and poor membrane association (23% ± 7%) (Fig. 2C). To measure the effect of phosphomimicking mutations on budding in the context of the mini-MPR, which contains a single serine S43, we generated the NEC220Δ40-S43E-His_8_ mutant. The S43E mutation reduced budding (63% ± 9%) (Fig. 2B) while preserving efficient membrane association (63% ± 8%) (Fig. 2C), showing that adding a single negative charge to the UL31 mini-MPR impairs the budding ability of the NEC.

The location of basic clusters influences NEC membrane budding activity, so we hypothesized that the placement of phosphorylatable serines relative to basic residues also is important for inhibition. Within the HSV-1 UL31 MPR, the 14 basic residues fall into five distinct clusters (Fig. 2A), and each, except the C-terminal one, has at least one serine nearby (Fig. 2A). To investigate whether single serine-to-glutamate mutations per cluster would recapitulate the inefficient budding phenotype of NEC220-SE6-His_8_, we generated the S11E/S24E/S43E mutant (NEC220-SE3-His_8_). However, NEC220-SE3-His_8_ supported efficient budding (78% ± 15%) (Fig. 2B) and membrane association (70% ± 6%) (Fig. 2C), showing that while adding six negative charges was sufficient to inhibit budding, adding three was not. Thus, the budding ability of the NEC requires not only basic clusters but also a sufficiently high net positive charge within the UL31 MPR.

Collectively, these results show that phosphomimicking mutations within the UL31 MPR, which introduce negative charges, reduce its budding activity, which confirms the importance of the net positive charge within the UL31 MPR for the NEC function (Fig. 3). We propose that the impaired nuclear egress and reduced titers of the phosphomimicking HSV-1 NEC mutant *in vivo* (33) are due to its reduced budding activity. Phosphorylation, which also introduces negative charges, would be expected to have a similar inhibitory effect on budding. We hypothesize that by inhibiting the budding activity of the NEC, phosphorylation could serve to prevent unproductive budding prior to the arrival of the capsid (reviewed in reference 5).

### Soluble NEC inserts peripherally into tethered lipid bilayers.

**FIG 4:**
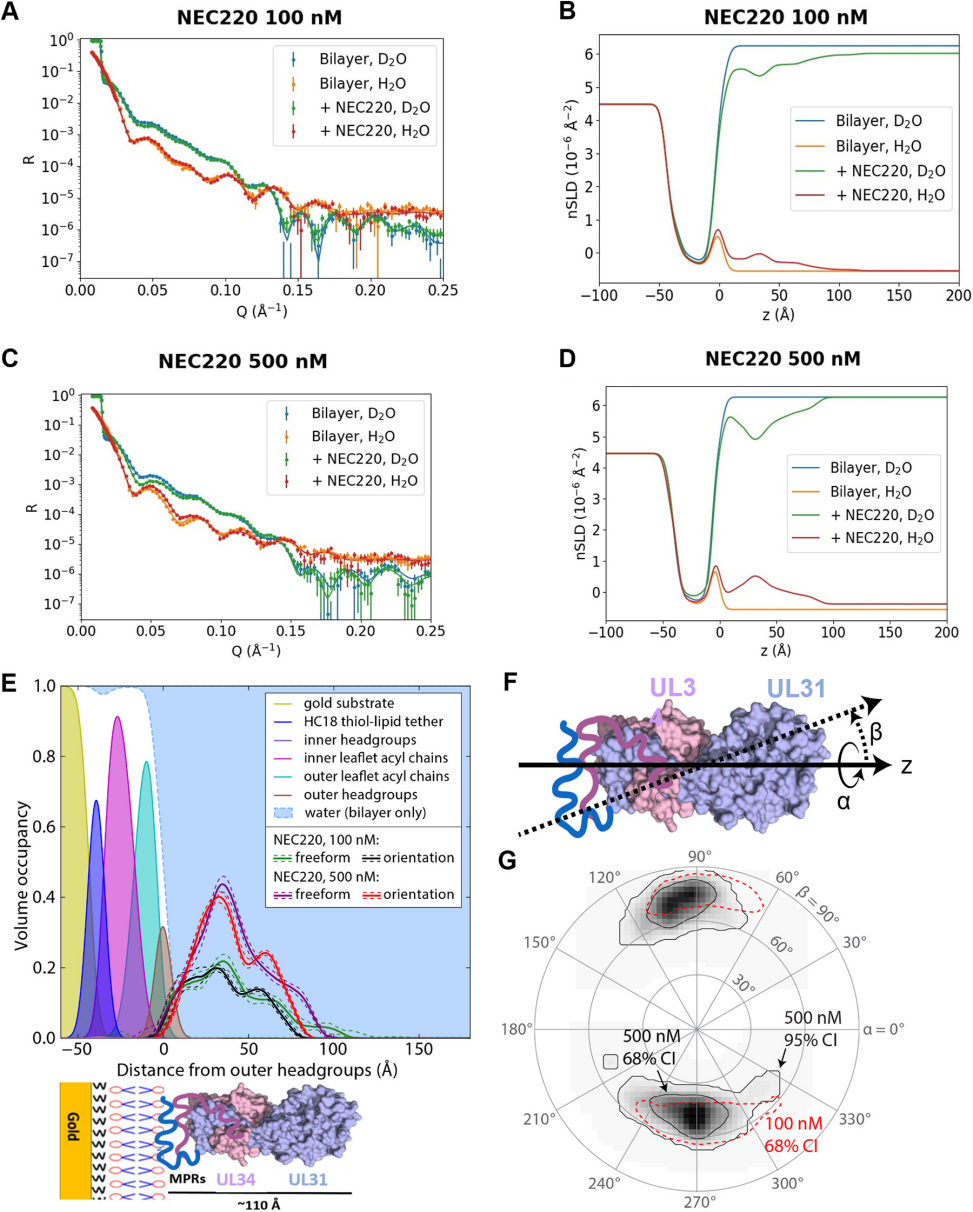
NEC inserts into polar lipid headgroups. (A and C) Neutron reflectivity for the prepared sparsely tethered lipid bilayer membrane on a gold thin-film substrate before and after incubation with NEC220 at the indicated solution concentration. Measurements were performed in both D_2_O- and H_2_O-based buffers to provide contrast. Solid curves are calculated from composition space model optimized to the experimental data. (B and D) Neutron scattering length density (nSLD) profiles corresponding to the solid lines in panels A and C and calculated from the composition space models. The gold substrate is at the left, and the buffer at the right, of each plot. The coordinate *z* is taken to be the distance above the center of the outer headgroups of the bilayer. (E) Volume density profiles of 3:1:1 mol% POPC:PS:PA lipid membranes determined from fitting a composition space model to the NR spectra. Density profiles of substrate and bilayer components are shown by filled curves; the sum is shown by the dashed blue line, and water fills the remaining space. Protein density profiles derived from freeform (Catmull-Rom spline) and orientation (Euler rotations of the crystallographic structure) models after incubation with 100 nM and 500 nM bulk concentrations of NEC220 and subsequent buffer rinses. Dashed lines indicate 68% confidence intervals on the protein density profiles. Schematic underneath graph is shown to provide context for each peak in graph. (F) Euler angle rotation scheme. (G) Probability plot for the orientation of NEC220 at the membrane as parameterized by the Euler angles α and β shown in panel F. The contour lines represent the 68% and 95% confidence intervals, as labeled.

To determine the orientation of the NEC on the membrane and how deeply it inserts into the lipid bilayer, we turned to neutron reflectometry (NR) (43), which allows low-resolution structural characterization of the membrane and any associated protein. A tethered lipid bilayer composed of POPC/POPS/POPA in a 3:1:1 molar ratio was prepared in a flow cell, and the reflectivity and scattering length densities of the bilayer interface to a collimated neutron beam, incident at various angles, was measured before and after incubation with NEC220 at 100 nM or 500 nM (Fig. 4A to D). Protein density profiles calculated from the NR measurements at each NEC concentration overlapped only the density profile of the outer lipid headgroup (Fig. 4E), suggesting that NEC220 inserted only into the polar lipid headgroup region, without penetration of large domains into the acyl chain region.

Within the NEC coats formed *in vitro* and *in vivo*, the NEC molecules are oriented perpendicularly to the plane of the membrane, with the protein density extending ∼110 Å from the membrane, in accordance with the cryo-EM measurements (21, 25). However, the NEC220 density profile obtained from the NR measurements only extended to ∼90 Å, and an orientation probability plot showed significant tilt of NEC220 from a vertical orientation (Fig. 4F and G). These data suggest that on the NR substrates, NEC220 adopts a tilted orientation relative to the plane of the membrane. However, because NR data are averaged over both time and space, they likely reflect different states of the NEC, characterized by different levels of positional freedom, for example, individual heterodimers versus higher oligomers. We hypothesize that whereas the individual NEC heterodimers can adopt a range of orientations relative to the plane of the membrane, oligomerization into hexagonal patches, or even individual hexamers, would restrict the movement of the NEC molecules, resulting in a more upright NEC density profile. We note that the intrinsic flatness of the NR substrates, or, alternatively, the underlying grain structure of the gold, may prevent the formation of extended hexagonal coats.

We also observed that after exposure to 500 nM NEC220, which deposited NEC220 on the membrane surface at high density (protein/lipid [P/L] molar ratio of 1:45), the membrane thickened by 0.49 ± 0.17 Å (68% confidence interval) in the context of the orientation model (see Table S1 in the supplemental material). Thinning of membranes tethered to flat substrates has been observed with proteins that generate positive curvature by inserting into the headgroup region (44–46). This is because forcing the headgroups apart on a flat substrate increases the area per lipid and thins the membrane (the hydrophobic tails form a constant-volume cylinder, the height of which must decrease if the area is increased). Conversely, membrane thickening could occur if the headgroups were forced closer together on a flat substrate, which, on free membranes, would result in negative mean membrane curvature. We hypothesize that the ability of the NEC to generate negative membrane curvature manifests as membrane thickening on the NR substrates.

10.1128/mBio.01548-21.8 TABLE S1 Parameters of the NR orientation model for 500 nM NEC220. ^a^, For clarity, parameters for the Catmull-Rom freeform spline model are not shown. ^b^, 68% confidence intervals are calculated from the stable solution to the MCMC optimization. ^c^, Based on control measurement of bilayer alone. ^d^, Defined in the model as the change in the inner lipid leaflet thickness. ^e^, The two Euler angles a and b and the protein depth are highly correlated and are not reported as a value with standard errors. See Fig. 3E and G. ^f^, The MPR was constrained to be near to the globular part of the protein. This parameter ranges from 1 (full extension of the MPR from the globular part of the protein) to 0 (full overlap of the MPR with the globular domains). Download Table S1, PDF file, 0.05 MB. Copyright © 2021 Thorsen et al. 2021 Thorsen et al. https://creativecommons.org/licenses/by/4.0/ This content is distributed under the terms of the Creative Commons Attribution 4.0 International license.

### NEC UL31 MPR peptides induce lipid headgroup ordering.

**FIG 5:**
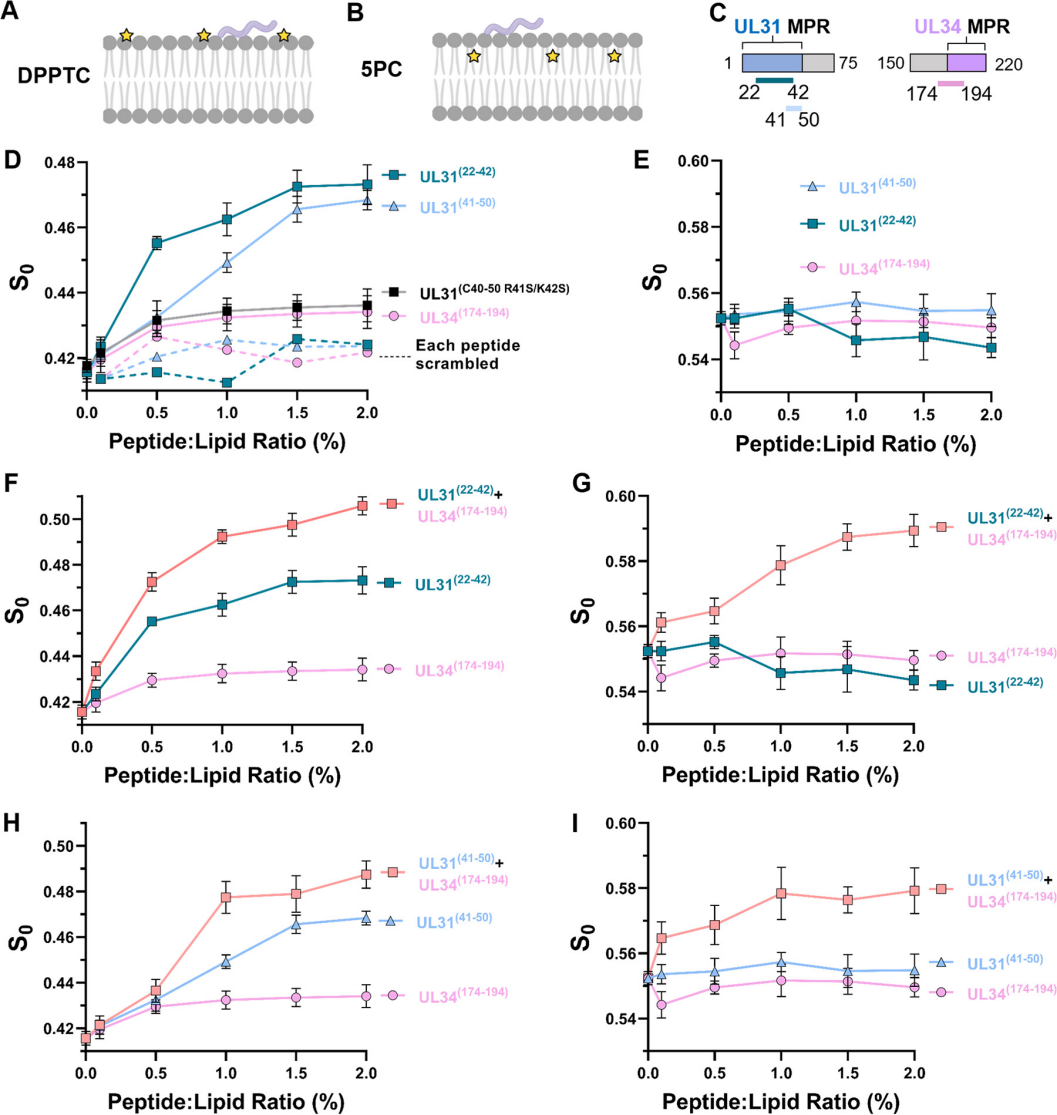
UL31 and UL34 membrane-proximal region peptides induce lipid headgroup and acyl chain ordering. (A) Schematic depicting DPPTC (yellow star is the probe) spin-labeled lipid in membrane and peptide (light purple). Image created with BioRender.com. (B) Schematic depicting 5PC spin-labeled lipid in membrane with peptide. Image created with BioRender.com. (C) Schematic depicting peptide location within the NEC sequence. (D) Plot of order parameters of DPPTC in POPC/POPS/POPA 3:1:1 versus the P/L ratio of individual UL31^(22–42)^ (teal), UL31^(41–50)^ (light blue), UL34^(174–194)^ (light pink), and UL31^R41S/K42S(41–50)^ (black). Scrambled peptides shown with dashed lines, color coded as previously stated. In all plots, error bars represent standard deviation (68% confidence interval of the data) of at least three individual experiments. (E) Plot of order parameters of 5PC in POPC/POPS/POPA 3:1:1 versus the P/L ratio of individual UL31^(22–42)^, UL31^(41–50)^, and UL34^(174–194)^. (F) Plot of order parameters of DPPTC in POPC/POPS/POPA 3:1:1 versus the P/L ratio of individual UL31^(22–42)^, UL34^(174–194)^, and combination of the two (light orange). (G) Plot of order parameters of 5PC in POPC/POPS/POPA 3:1:1 versus the P/L ratio of UL31^(22–42)^, UL34^(174–194)^, and combination of the two. (H) Plot of order parameters of DPPTC in POPC/POPS/POPA 3:1:1 versus the P/L ratio of individual UL31^(41–50)^, UL34^(174–194)^, and combination of the two. (I) Plot of order parameters of 5PC in POPC/POPS/POPA 3:1:1 versus the P/L ratio of individual UL31^(41–50)^, UL34^(174–194)^, and combination of the two.

To determine how the MPRs influence the structure of the lipid bilayer, we turned to continuous-wave electron spin resonance (CW-ESR) using spin-labeled lipids, which generate an ESR signal. The spin-labeled lipid within the membrane is sensitive to the local environment, and, therefore, the ESR signal reports on the mobility of the spin label, which, in turn, reports on the order of the lipids in the membranes. The order parameter of the spin (*S*_0_), which is calculated from the CW-ESR spectra, correlates with the local lipid order and inversely correlates with the mobility of the spin label. Thus, the effect of peptide binding on the lipid order can be monitored. Two phosphatidylcholine derivatives containing spin labels were used: dipalmitoylphosphatidyl-tempo-choline (DPPTC), which has a tempo-choline headgroup with a spin sensitive to the environment within the headgroup region (Fig. 5A), and 1-palmitoyl-2-stearoyl-(5-doxyl)-sn-glycero-3-phosphocholine, which has a doxyl group in the C-5 position of the acyl chain where the spin is sensitive to the environment within the upper acyl chain (Fig. 5B). These two spin-labeled lipids were used in previous studies, which validated their ability to detect changes in lipid order (47–55).

To investigate the effect of the NEC MPRs on lipid order, we used three UL31-derived peptides: UL31^(41–50)^, which corresponds to the mini-MPR; UL31^(C40–50 R41S/K42S)^, which corresponds to the mini-MPR with the mutated N-terminal dibasic motif and contains an N-terminal cysteine for spin-labeling in later experiments (Fig. 2); and UL31^(22–42)^, which corresponds to the middle of the UL31 MPR. We also used one UL34-derived peptide, UL34^(174–194)^, which encompasses a portion of the UL34 MPR (Fig. 5C). The boundaries of UL31^(22–42)^ and UL34^(174–194)^ were chosen using a machine-learning classifier that identifies peptide sequences with the capacity to generate negative Gaussian curvature in membranes, which is topologically required in membrane-remodeling processes such as membrane budding and fission (56). As controls, we also prepared scrambled versions of the peptides: UL31^scr(41–C51)^, UL31^scr(22–C43)^, and UL34^scr(174–194)^. Scrambled UL31 peptides contained C-terminal cysteines for spin-labeling in later experiments. Peptide sequences are listed in Table S2.

10.1128/mBio.01548-21.9 TABLE S2 Sequences of UL31 and UL34 peptides used in CW-ESR experiments (Fig. 5). Download Table S2, PDF file, 0.02 MB. Copyright © 2021 Thorsen et al. 2021 Thorsen et al. https://creativecommons.org/licenses/by/4.0/ This content is distributed under the terms of the Creative Commons Attribution 4.0 International license.

If peptide binding to the membrane increases the mobility of the spin-labeled probe, we would expect to see a decrease in the order parameter, *S*_0_, with increasing peptide/lipid (P/L) ratio. Conversely, if peptide binding decreases the mobility of the spin-labeled probe, we would see an increase in *S*_0_ (47). All three native peptides UL31^(41–50)^, UL31^(22–42)^, and UL34^(174–194)^ increased the *S*_0_ in the headgroup region (DPPTC) (Fig. 5D) in a sequence-specific manner, with the native UL31 peptides inducing significantly larger lipid headgroup ordering than the scrambled versions. However, none of the individual MPR peptides induced obvious ordering of the upper acyl chain (5PC) (Fig. 5E). The CW-ESR experiments also showed that the UL31^(C40–50 R41S/K42S)^ mutant peptide, which lacks the N-terminal dibasic motif, induced substantially less lipid headgroup ordering than the WT UL31^(41–50)^ peptide (Fig. 5D).

Decreased lipid headgroup ordering by the UL31^(C40–50 R41S/K42S)^ and the UL31^scr(41–C51)^ peptides (Fig. 5D) correlates with the reduced budding activity of the respective mutant NEC constructs NEC220Δ40-R41S/K42S-His_8_ (34% ± 10%) and NEC220Δ40scr-His_8_ (11% ± 8%) (Fig. 2B). Decreased lipid headgroup ordering by the UL31^(C40–50 R41S/K42S)^ mutant peptide could be due to reduced membrane binding (relative to UL31^(41–50)^), as determined by the ESR partition ratio (Fig. S1). However, the UL31^scr(41–C51)^ peptide binds membranes similarly to UL31^(41–50)^ (Fig. S1), so the observed decrease in lipid headgroup ordering could not be due to impaired membrane interactions. These results suggest that both lipid ordering (Fig. 5D) and efficient budding *in vitro* (Fig. 2B) require not only the +4-net charge but also charge clusters, namely, 2 dibasic motifs.

10.1128/mBio.01548-21.1 FIG S1 Membrane partition ratios of NEC MPR peptides. The partition ratio of the N- and C-terminal spin labeled UL31 and UL34 MPRs in the presence of POPC/POPS/POPA=3/1/1 SUV membranes. The partition ratios were calculated from the amount of spins in the supernatant and pellet of the double integral of the ESR signal. The averages and standard deviations (68% confidence intervals of the data) were calculated from three independent experiments. Download FIG S1, PDF file, 0.06 MB. Copyright © 2021 Thorsen et al. 2021 Thorsen et al. https://creativecommons.org/licenses/by/4.0/ This content is distributed under the terms of the Creative Commons Attribution 4.0 International license.

### In combination, UL31 and UL34 MPR peptides induce both lipid headgroup and acyl chain ordering.

We next examined how a combination of UL31 and UL34 MPR peptides would affect lipid order. A mixture of UL31 and UL34 MPR peptides at a 1:1 molar ratio was mixed with liposomes containing spin-labeled lipids in various P/L ratios. When comparing *S*_0_ at the same P/L ratio, the UL31^(22–42)^/UL34^(174–194)^ combination increased the local order in the headgroup region (DPPTC) to a greater extent than the individual peptides alone (Fig. 5D and F). The same effect was observed for the UL31^(41–50)^/UL34^(174–194)^ combination (Fig. 5D and H). Moreover, both the UL31^(22–42)^/UL34^(174–194)^ and the UL31^(41–50)^/UL34^(174–194)^ combinations induced ordering of the upper acyl chains (5PC; Fig. 5G and I), in contrast to the individual peptides (Fig. 5E). Therefore, while individually UL31 and UL34 MPR peptides induce lipid headgroup ordering, in combination they induce greater lipid headgroup ordering as well as the ordering of the upper acyl chains. Thus, the UL31 and UL34 MPR peptides act cooperatively.

The ESR measurements were also performed with NEC220 and NEC220Δ40. Both protein complexes induced membrane ordering in the headgroup region, with NEC220 having a larger effect than the NEC220Δ40 (Fig. S2A). The “nominal” P/L ratio of the complex required to saturate the *S*_0_-P/L ratio curve was significantly larger than that of the peptide mixtures, which could be due to the different binding constants of the peptides relative to the NEC constructs. The ESR experiments utilized small unilamellar vesicles (SUVs), <100 nm in diameter, which both NEC220-His_8_ and NEC220Δ40-His_8_ bind less efficiently than lipid vesicles of larger size (Fig. S2B). Thus, the CW-ESR results show that both the MPR-derived peptides and the NEC can induce membrane ordering.

10.1128/mBio.01548-21.2 FIG S2 NEC220 and NEC220Δ40 induce lipid headgroup ordering and bind well to vesicles with less curvature. (A) Membrane ordering of DPPTC induced by NEC220 (black) and NEC220Δ40 (red). Error bars show the standard deviation (68% confidence interval of the data) from 3 independent experiments. (B) Purified NEC and vesicles of the indicated sizes containing 40% negatively charged lipids were co-floated by ultracentrifugation. Larger vesicles have smaller degrees of curvature. Each bar represents the amount of protein floating with vesicles in the top fraction minus background levels of floating protein in the absence of vesicles. Significance was calculated to binding of 100-nm vesicles for each respective construct using an unpaired Student’s *t*-test with Welch’s correction (*P* < 0.05 = *). Error bars represent the standard error of the mean (68% confidence interval of the mean) for at least two individual experiments. Download FIG S2, PDF file, 0.2 MB. Copyright © 2021 Thorsen et al. 2021 Thorsen et al. https://creativecommons.org/licenses/by/4.0/ This content is distributed under the terms of the Creative Commons Attribution 4.0 International license.

### In the presence of the UL34 MPR, the UL31 MPR inserts more deeply into the membrane.

To measure how deeply the MPR peptides insert into the membrane, we performed power saturation ESR (57–59) with peptides spin-labeled with S-(1-oxyl-2,2,5,5-tetramethyl-2,5-dihydro-1H-pyrrol-3-yl) (MTSL) on either an N-terminal cysteine [UL31^(C40–50)^ and UL31^(C21–42)^] or a C-terminal cysteine [UL31^(41–C51)^ and UL31^(22–C43)^]. The depth of spin label insertion into the membrane was determined from the accessibility of each peptide to O_2_, which penetrates into the hydrophobic region of the membrane, or Ni(II)‐diammine‐2,2′‐(ethane‐1,2‐diyldiimino) diacetic acid (NiEDDA), which does not penetrate the membrane beyond the polar headgroup region. The insertion depth parameter Φ, which represents the difference in the accessibility of the spin label to O_2_ versus NiEDDA, reports on the spin label insertion depth, with Φ = 0 corresponding to the hydrophobic/hydrophilic interface. Thus, the more positive the Φ, the deeper the residue inserts into the hydrophobic core, whereas a negative Φ means the residue remains in the polar headgroup region.

**FIG 6:**
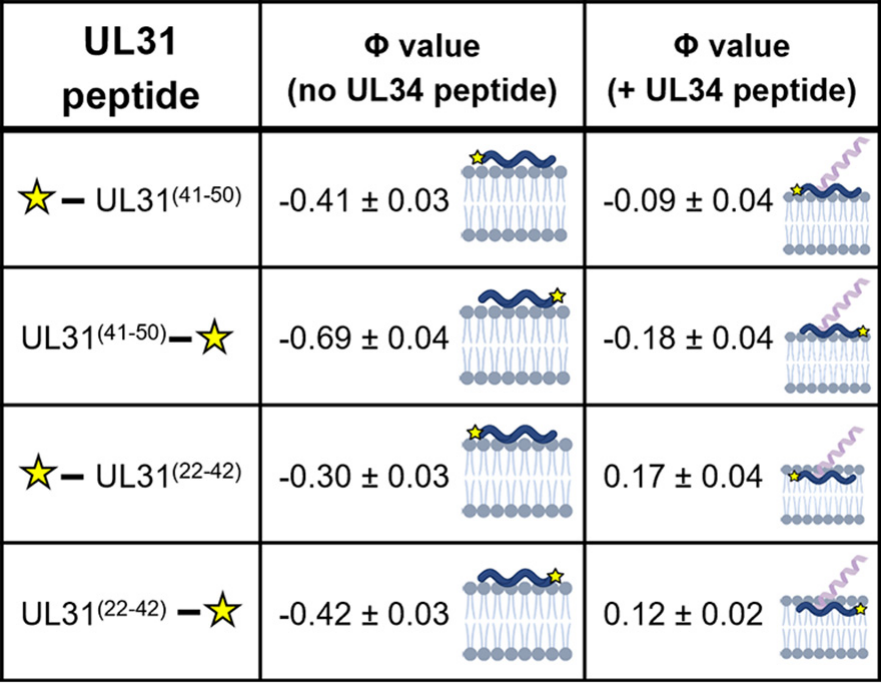
Presence of the UL34 peptide deepens membrane penetration of UL31 MPR peptides. The insertion depth parameter Phi (Φ) values of the N- and C-terminal spin labeled UL31 MPRs in the presence of POPC/POPS/POPA 3:1:1 SUVs. The phi values were calculated from the power saturation ESR spectra. The averages and standard deviations (68% confidence intervals of the data) were calculated from three independent experiments. Schematics to the right of Φ values depict probe (yellow star) placement on peptides and roughly estimated insertion depths. Images created with BioRender.com.

The Φ values for the spin-labeled UL31^(C40–50)^ and UL31^(41–C51)^ were −0.41 ± 0.03 (68% confidence interval) and −0.69 ± 0.04, respectively (Fig. 6), which indicated that they both reside in the lipid headgroup region. However, when the spin-labeled UL31^(C40–50)^ and UL31^(41–C51)^ peptides were mixed with the unlabeled UL34^(174–194)^ peptide at a 1:1 molar ratio, the Φ values increased to −0.09 ± 0.04 and −0.18 ± 0.04, respectively, consistent with a deeper insertion into the hydrophobic/hydrophilic interface (Fig. 6). A similar trend was observed for UL31^(C21–42)^ and UL31^(22–C43)^, where the Φ values of the spin-labeled cysteines increased from −0.30 ± 0.03 and −0.42 ± 0.03 to 0.17 ± 0.04 and 0.12 ± 0.02, respectively, in the presence of the unlabeled UL34^(174–194)^ peptide (Fig. 6). The power saturation ESR results suggest that the UL31 MPR inserts more deeply into the membrane in the presence of UL34 MPR. This observation complements the CW-ESR results, which showed that the 1:1 mix of UL31 and UL34 MPR peptides induces lipid ordering within the upper acyl chains (Fig. 5G and I), which could result from the deeper insertion of the UL31 MPR into the upper acyl chain region in the presence of the UL34 MPR. Alternatively, the UL31 MPR may remain in the lipid headgroup region while drawing the headgroups together and thereby constraining the motion of the upper acyl chains and the spin label located there.

### UL31 and UL34 MPRs induce negative Gaussian curvature in membranes.

**FIG 7:**
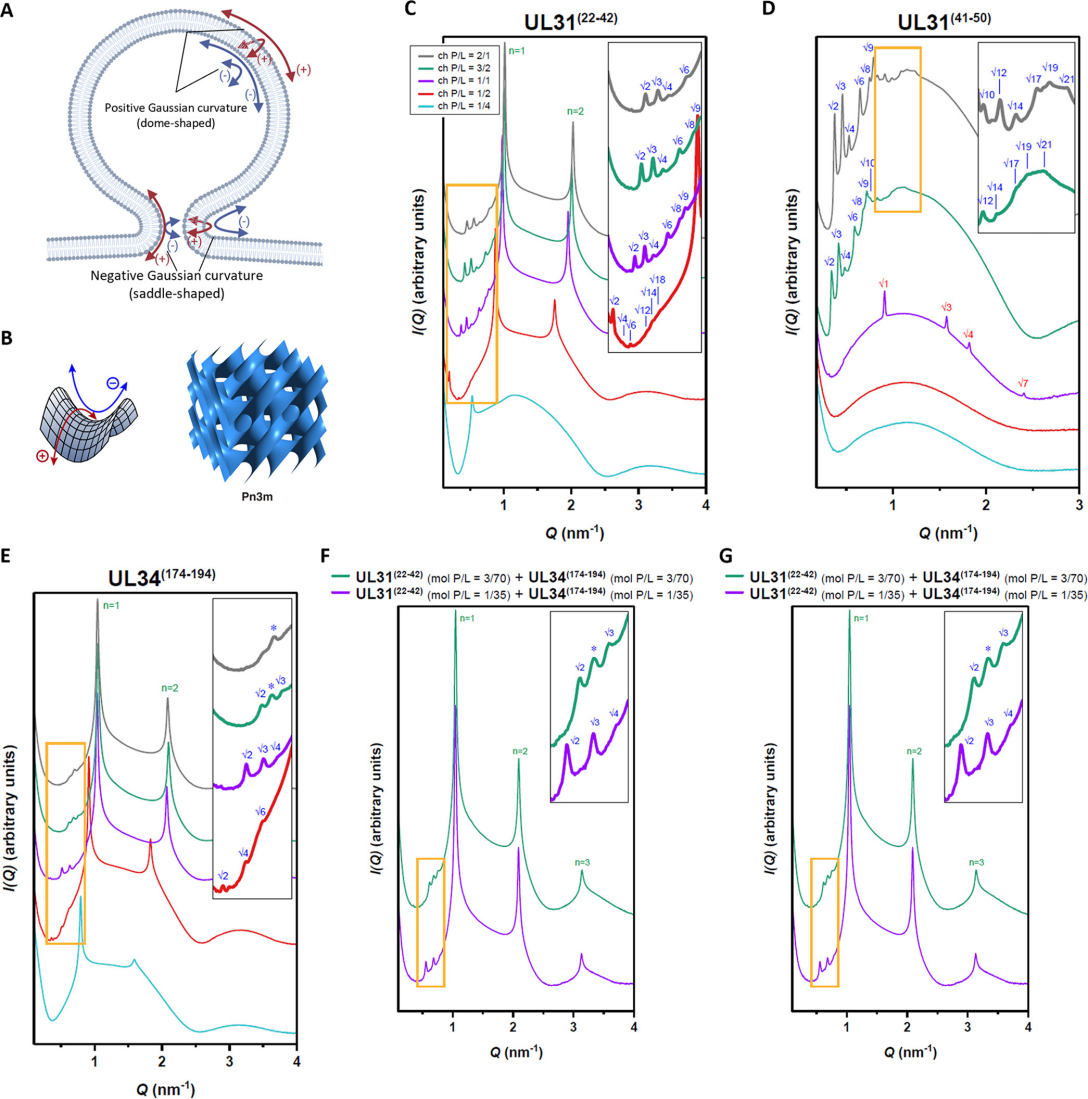
UL31 and UL34 membrane-proximal region peptides generate negative Gaussian curvature in membranes. (A) Schematic depicting the principal curvatures of the neck of a budding vesicle which together generate negative Gaussian curvature. Image created with BioRender.com. (B) A saddle-shaped surface (left) is described by negative Gaussian curvature, which requires positive curvature in one direction and negative curvature in the orthogonal direction. Lipids can exhibit diverse phases, including cubic phases. A bicontinuous cubic phase, such as Pn3m (right), consists of a curved membrane bilayer with saddle surfaces that follow the geometry of a periodic minimal surface. Negative Gaussian curvature is found at every point along the surfaces of cubic phases. Reflections from cubic phases (labeled in blue) are observed in the SAXS spectra for SUVs incubated with individual peptides (C) UL31^(22–42)^, (D) UL31^(41–50)^, (E) UL34^(174–194)^, and two combinations of approximately equimolar amounts of each UL31 and UL34 MPR peptide (F) UL31^(22–42)^/UL34^(174–194)^, and (G) UL31^(41–50)^/UL34^(174–194)^. (C to G) For improved visualization, spectra have been manually offset in the vertical direction by scaling each trace by a multiplicative factor. For clarity, the insets show expanded views of the lower intensity cubic reflections (orange-boxed regions). Indexed reflections from coexisting hexagonal (red) and lamellar (green) phases are also labeled. Asterisks denote peaks that could not be indexed to a phase due to absence of higher order reflections.

To determine the effect of the MPRs on membrane curvature, we used small-angle X-ray scattering (SAXS) to quantitatively characterize membrane deformations upon exposure to MPR-derived peptides UL31^(22–42)^, UL31^(41–50)^, UL34^(174–194)^, and their combinations. SAXS can detect the generation of negative Gaussian curvature (NGC) (60–63), which corresponds to the saddle-like curvature found on the inside a donut hole, the inner surface of membrane pores, and the necks of budding vesicles (Fig. 7A) and is required for membrane-remodeling events such as vesicle budding (63), membrane fission (64), membrane fusion (65), and pore formation (62). In contrast, positive Gaussian curvature (PGC) corresponds to the dome-like curvature such as found on a spherical body of the bud (Fig. 7A).

SUVs with a 1:4 molar ratio of DOPS/DOPE were incubated with each peptide or a combination of peptides at peptide-to-lipid charge ratios (ch P/L) of 1:4, 1:2, 1:1, 3:2, and 2:1 (see Materials and Methods for the equivalent peptide-to-lipid molar ratios [mol P/L]) and measured using SAXS. We choose a lipid composition DOPS/DOPE ratio of 20:80 because it has a surface charge density typical of eukaryotic membranes and can sense the capacity for the induction of membrane curvature, including NGC. The induction of NGC was monitored by the appearance of correlation peaks that correspond to NGC-rich *Im3m* and *Pn3m* cubic phases, which are defined by a lattice parameter *a* and an average NGC |<*K*>| (Fig. 7B to G and Fig. S3B to F). Both *Im3m* and *Pn3m* are inverse bicontinuous cubic phases (Q_II_), which are lyotropic liquid-crystalline phases that can be formed by lipid systems. A bicontinuous cubic phase consists of two interpenetrating, but nonintersecting, aqueous volumes that are separated by a single continuous lipid bilayer. The mid-plane of this bilayer traces a minimal surface that is characterized by having NGC at all points on its surface.

10.1128/mBio.01548-21.3 FIG S3 SAXS spectra for control and indexing of the identified liquid-crystalline lipid phases. (A) Spectra for SUVs in buffer, individual peptides in buffer, and buffer-only controls. For improved visualization, spectra have been manually offset in the vertical direction by scaling each trace by a multiplicative factor. Linear regressions of indexed reflections for phases induced by (B) UL31^(22-42)^, (C) UL31^(41-50)^, (D) UL34^(174-194)^, (E) UL31^(22-42)^/ UL34^(174-194)^, and (F) UL31^(41-50)^/ UL34^(174-194)^. (B to F) Vertical dotted reference lines denote indexed reflections. Each plotted point corresponds to an indexed peak, with its associated lattice indicated by the marker symbol (circle = *Im3m* cubic, diamond = *Pn3m* cubic, triangle = hexagonal). Colors of each regression correspond to the same ch P/L values in Fig. 7. Calculated lattice parameters and NGC values for the identified phases are provided in Fig. S4. Download FIG S3, PDF file, 0.3 MB. Copyright © 2021 Thorsen et al. 2021 Thorsen et al. https://creativecommons.org/licenses/by/4.0/ This content is distributed under the terms of the Creative Commons Attribution 4.0 International license.

While the SUVs alone displayed a broad characteristic feature consistent with the form factor expected for unilamellar vesicles (Fig. S3A), all three individual peptides and the two peptide mixtures restructured the membranes into NGC-rich cubic phases with different amounts of NGC (Fig. 7C to G and Fig. S3B to F and S4). The NGC magnitude generally increased with increasing peptide concentration. Among the three individual peptides, UL34^(174–194)^ induced the largest amounts of NGC on average, followed by UL31^(22–42)^ and UL31^(41–50)^. While all three individual peptides were able to form cubic phases, over five times the number of UL31^(41–50)^ peptide molecules were required to generate approximately the same quantitative amount of NGC as UL31^(22–42)^ or UL34^(174–194)^, which suggests that UL31^(41–50)^ peptide has a reduced capacity for NGC induction compared with the other two peptides.

Upon exposure to these peptides, in addition to the cubic phases, the membranes tended to form additional coexisting phases, which suggested the presence of other modes of membrane deformation. Interestingly, at ch P/L = 1:1, UL31^(41–50)^ formed an inverse hexagonal phase (H_II_), which is characterized by having negative mean curvature (but zero Gaussian curvature). This property is in line with the requirement of the UL31 MPR for budding (Fig. 1D). Additionally, both UL31^(22–42)^ and UL34^(174–194)^ but not UL31^(41–50)^ induced coexisting lamellar phases (L_α_) (Fig. S4), but the relevance of these to the topological changes that occur during budding, if any, is unclear.

10.1128/mBio.01548-21.4 FIG S4 Phase diagram with tabulated lattice parameters and negative Gaussian curvature values for each SAXS condition. Cells containing dashed lines indicate that the phase was either not present or could not be indexed due to insufficient number of reflections. Download FIG S4, PDF file, 0.3 MB. Copyright © 2021 Thorsen et al. 2021 Thorsen et al. https://creativecommons.org/licenses/by/4.0/ This content is distributed under the terms of the Creative Commons Attribution 4.0 International license.

We further examined the membrane curvature effects of peptide combinations, UL31^(22–42)^/UL34^(174–194)^ and UL31^(41–50)^/UL34^(174–194)^. At approximately equimolar ratios, both peptide pairs generated higher magnitudes of NGC than the individual peptides (Fig. 7F and G and Fig. S3E and F and S4), demonstrating a cooperative effect between the UL31 and UL34 MPR peptides, which is consistent with their cooperativity in inducing lipid ordering observed by the ESR. This is also consistent with previous studies that showed that embedded peptides and proteins introduce intramembrane stresses and strains that lead to negative curvature generation and alter membrane bending stiffness (66, 67). Thus, while the UL31 and UL34 MPR peptides can generate NGC as individual peptides, they do so more effectively when combined. Using a catenoid surface model (63, 64, 68), we estimated the size of the constricted membrane neck of a budding vesicle that can be formed from the largest amount of NGC induced by the UL31 and UL34 MPR peptides to be |<*K*>| = 3.21 × 10^−2 ^nm^−2^, which corresponds to a membrane neck with an inner diameter of 7.2 nm and an outer diameter of 15.2 nm (assuming an ∼4-nm-thick membrane bilayer). This estimate is in agreement with the diameters of the scission necks formed by mitochondrial fission proteins (64) and with the theoretical calculations (68). Thus, the MPRs can generate membrane curvature necessary for neck scission, which is consistent with the NEC-induced bud scission observed *in vitro* (21).

### DEER analysis suggests that UL31 and UL34 MPRs interact on membranes.

The cooperative effect of the UL31 and the UL34 MPR peptides on lipid ordering and induction of NGC as well as a greater depth of insertion of the UL31 MPR peptides in the presence of UL34 MPR peptide suggest that the UL31 and UL34 MPR peptides interact. To determine if the UL31 and UL34 peptides interact on membranes, we employed double electron-electron resonance (DEER) spectroscopy, which yields the distance distributions between two spin systems in a frozen sample and is sensitive within the 20- to 80-Å range (69, 70). The recently developed pulse-dipolar electron spin resonance spectroscopy wavelet denoising methodology removes the noise from the ESR spectra and improves their accuracy (71), thereby reducing the uncertainty in distance distribution reconstruction by a special singular value decomposition methodology (72, 73).

In our experiments, one spin was attached to either the N- or the C-terminal cysteine of a UL31 peptide and the other, to the native cysteine, C182, of the UL34 MPR peptide. None of the individual peptides exhibited any DEER signal in the presence of SUVs at a 1:200 P/L ratio [see representative DEER spectra of UL31^(C1–50)^ and UL34^(174–194)^ in Fig. S5A and B]. In a spin echo control experiment, strong spin echoes were observed (Fig. S5C), which indicated that the peptides were properly spin-labeled and did not aggregate, ruling out the possibility that the phase memory time (*T_m_*) was too short to observe the DEER signal. Therefore, the lack of a DEER signal with individual peptides indicates that they do not homodimerize in the presence of SUVs (74).

10.1128/mBio.01548-21.5 FIG S5 DEER and spin echo signals. (A) DEER signal for UL31^(C1-50)^ in membranes. (B) DEER signal for UL34^(174-194)^ in membranes. (C) Spin echo signal for UL31^(C1-50)^ in membranes. (D) DEER signal for UL31^(C1-50)^/UL34^(174-194)^ in solution. Download FIG S5, PDF file, 0.03 MB. Copyright © 2021 Thorsen et al. 2021 Thorsen et al. https://creativecommons.org/licenses/by/4.0/ This content is distributed under the terms of the Creative Commons Attribution 4.0 International license.

**FIG 8:**
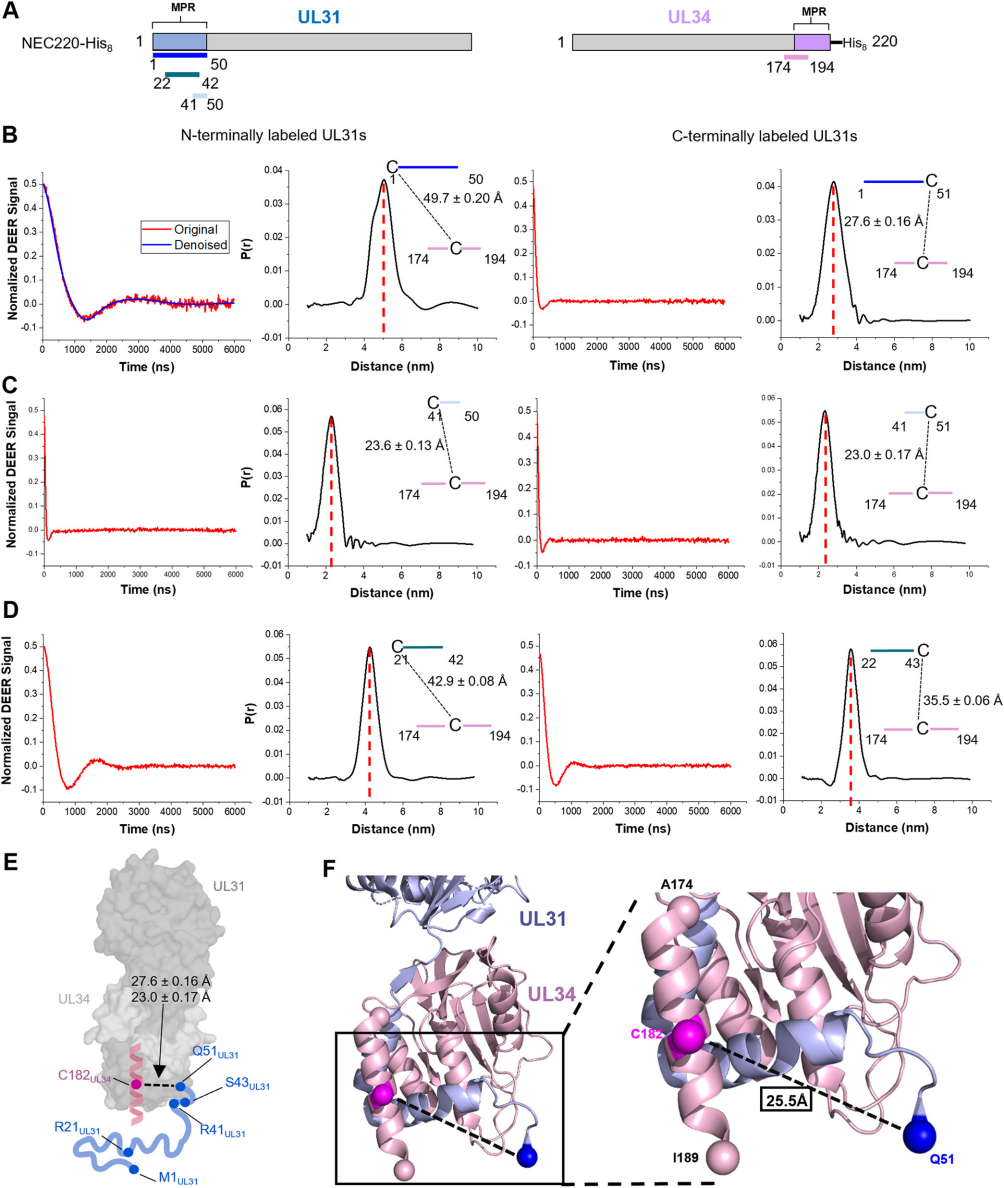
UL31 and UL34 MPR peptides interact in the presence of membranes. (A) Schematic depicting peptide location within the NEC sequence. (B, C, and D) Representative experimental DEER data (left) and reconstructed interpeptide distance distributions (right) of at least two individual experiments. The “C” indicates the location of spin-labeled Cys. (B) UL31^(C1–50)^/UL34^(174–194)^ and UL31^(1–C51)^/UL34^(174–194)^. The blue line in the left panel is the denoised curve. Schematic depicts interpeptide distance in Ångstroms and associated standard error of the mean (68% confidence interval of the mean) for each tested probe location. (C) UL31^(C40–50)^/UL34^(174–194)^, UL31^(41–C51)^/UL34^(174–194)^, (D) UL31^(C21–42)^/UL34^(174–194)^, or UL31^(22–C43)^/UL34^(174–194)^. The peptides were mixed with POPC/POPS/POPA 3:1:1 SUVs in 1:200 P/L ratio. Each combination was repeated two times. (E) Model of HSV-1 UL31 and UL34 MPRs attached to greyscale HSV-1 NEC crystal structure based on DEER measurements. Native amino acid sequences were substituted for cysteine probe locations and are shown as filled in circles. UL34 MPR is alpha helical in homologous structures and depicted as such, while UL31 is shown as unstructured. (F) Bottom of homology-modeled HSV-1 NEC onto PRV NEC. Inset shows distance measurement between Q51_UL31_ and C182_UL34_ shown as spheres. A174_UL34_ and I189_UL34_ are shown as spheres. Images taken in PyMoL.

Next, we mixed each of the six singly labeled UL31 MPR peptides [UL31^(C1–50)^, UL31^(1–C51)^, UL31^(C40–50)^, UL31^(41–C51)^, UL31^(C21–42)^, or UL31^(22–C43)^] with the singly labeled UL34 MPR peptide at a 1:1 ratio in the presence of SUVs. DEER measurements between UL34^(174–194)^ and UL31^(1–C51)^ or UL31^(41–C51)^ were similar, 27.6 ± 0.16 Å (68% confidence interval of the mean) and 23.0 ± 0.17 Å, respectively (Fig. 8B, C, and E), which suggests that the mini-MPR recapitulates the interactions of the full-length UL31 MPR. Additionally, residues C40_31_ and C51_31_ are equidistant from C182_34_ (23.6 ± 0.13 Å and 23.0 ± 0.17/27.6 ± 0.16 Å, respectively), whereas both C21_31_ and C1_31_ are much farther away (42.9 ± 0.08 Å and 49.7 ± 0.20 Å, respectively) (Fig. 8B to E). This suggests that the UL31 MPR C terminus is closer to the UL34 MPR than its N terminus and likely interacts with it. The C43_31_-C182_34_ distance (35.5 ± 0.06 Å) is unexpectedly longer than both the C40_31_-C182_34_ and the C51_31_-C182_34_ distances (23.6 ± 0.13 Å and 23.0 ± 0.17/27.6 ± 0.16 Å, respectively) (Fig. 8B to E), but this could be due to the relative orientations of the spins, which are ∼6-Å away from the Cα (75). As a control, no DEER signal was observed in the absence of SUVs (Fig. S5D).

A common way to evaluate the DEER distance measurements is to compare them to the corresponding measurements in the high-resolution protein structures. Although the residues labeled in the DEER experiments were absent from the HSV-1 NEC structure (27), residues corresponding to 51 to 54 of UL31 and 177 to 189 of HSV-1 NEC were resolved in the crystal structure of the PRV NEC (27) and were modeled onto the HSV-1 NEC structure (Fig. 8F). The distance between Q51_34_ (Cα) and C182_34_ (Cα) in the model was 25.5 Å (Fig. 8F), which is similar to the DEER distances of 23.0 ± 0.17 Å and 27.6 ± 0.16 Å. Thus, the DEER results obtained with the MPR peptides are relevant to the MPRs in the context of the NEC.

### Chemical cross-linking confirms that UL31 and UL34 MPRs interact on membranes.

To confirm UL31/UL34 MPR interactions identified by DEER, we performed chemical cross-linking. The UL31 peptides have a primary amine at K42_31_ and no sulfhydryls, whereas the UL34 peptide has a sulfhydryl at C182_34_ and no primary amines, so the heterobifunctional SM(PEG)_6_ cross-linker that reacts with primary amines and sulfhydryls was used. SM(PEG)_6_, which can cross-link primary amines and sulfhydryls within 32.5 Å, should be capable of bridging the ∼30-Å distance between K42_31_ and C182_34_, as measured by DEER. The UL31^(41–50)^/UL34^(174–194)^ and UL31^(22–42)^/UL34^(174–194)^ combinations were only cross-linked in the presence of SUVs, whereas individually, UL31^(41–50)^ or UL34^(174–194)^ did not get cross-linked and UL31^(22–42)^ showed only low levels of cross-linking in the presence or absence of SUVs (Fig. S6), in agreement with the DEER data showing individual peptides do not form homodimers either in solution or on SUVs. The cross-linking results further establish that the peptides derived from the MPRs of UL31 and UL34 interact on the membranes.

10.1128/mBio.01548-21.6 FIG S6 Crosslinking of UL31 and UL34 MPR peptides. (A) Individual and combination of peptides were crosslinked in the absence or presence of 3/1/1=POPC/POPS/POPA SUVs in two individual experiments. SM(PEG)_6_ was added at 50-fold molar excess. Samples were analyzed by 16% Tricine-SDS-PAGE and Coomassie staining. Samples were run on gels grouped by presence or absence of SUVs. Images depict representative gels from one experiment where lanes are cropped to re-group based on peptides used rather than presence or absence of vesicles. Predicted molecular masses of individual peptides and 1:1 complexes are indicated. Red boxes denote crosslinked UL31-UL34 peptides. (B) Quantification of two individual experiments. Each bar represents the amount of peptide crosslinked. Error bars represent the standard error of the mean (68% confidence interval of the mean) for two individual experiments. Download FIG S6, PDF file, 0.1 MB. Copyright © 2021 Thorsen et al. 2021 Thorsen et al. https://creativecommons.org/licenses/by/4.0/ This content is distributed under the terms of the Creative Commons Attribution 4.0 International license.

### UL34 MPR peptide forms an α-helix in the presence of membranes.

Circular dichroism (CD) (76) was used to assess the secondary structure content of the UL31 and UL34 MPR peptides. A characteristic CD spectrum of an α helix has two negative troughs at 222 nm and 208 nm and a positive peak at 192 nm, whereas the CD spectrum of a random coil has low ellipticity above 210 nm and negative values near 195 nm (76, 77). The UL34^(174–194)^ was expected to form a helix because equivalent residues form α helices in the structures of PRV and HCMV UL34 homologs (27, 78–80). UL34^(174–194)^ peptide adopted a random-coil conformation in solution but became α-helical in the presence of SUVs (Fig. S7B), which suggested that its sequence has a propensity to form α-helical structure. In contrast, all UL31 peptides, UL31^(41–50)^, UL31^(22–42)^, or UL31^(1–50)^, adopted a random-coil conformation both in solution and in the presence of SUVs (Fig. S7C, E, and G). The spectra of the UL31^(41–50)^/UL34^(174–194)^ and UL31^(22–42)^/UL34^(174–194)^ combinations had helical signatures (Fig. S7D and F), but these were less pronounced than that of UL34^(174–194)^ alone (Fig. S7B), whereas the spectrum of the UL31^(1–50)^/UL34^(174–194)^ combination had no obvious helical signature (Fig. S7H). We hypothesize that the helical signature of UL31/UL34 peptide combinations is due to UL34 and is less pronounced than that of UL34^(174–194)^ due to the UL34 signal being “diluted” by the unstructured UL31 peptides. The CD data suggest that the UL31 MPR is unstructured even in the presence of UL34 MPR and membranes.

10.1128/mBio.01548-21.7 FIG S7 Circular dichroism of UL31 and UL34 MPR peptides. (A) Schematic illustrating peptide location in NEC sequence. UL34 174-194 sequence and predicted secondary structure shown underneath schematic. No part of UL31 MPR is predicted to form secondary structure. (B to H) Overlays of the far-UV CD spectra in the presence and absence of POPC/POPS/POPA 3:1:1 SUVs. Averages of five replicates are reported. Download FIG S7, PDF file, 0.3 MB. Copyright © 2021 Thorsen et al. 2021 Thorsen et al. https://creativecommons.org/licenses/by/4.0/ This content is distributed under the terms of the Creative Commons Attribution 4.0 International license.

## DISCUSSION

Generation of membrane curvature lies at the core of the membrane budding ability of the NEC, but how the NEC accomplishes this is unclear. Previous work has shown that the NEC oligomerizes into hexagonal scaffold-like coats on the inner surface of budded vesicles (21, 25, 26) and that oligomerization is essential for budding both *in vivo* and *in vitro* (21, 27, 28). Membrane scaffolding is a common mechanism for generating both positive and negative membrane curvature, e.g., by the BAR domain proteins (reviewed in reference 81) and HIV Gag (82), respectively. Therefore, one may conclude that formation of negative membrane curvature by the NEC is driven by scaffolding alone. However, here we show that highly basic MPRs of the NEC are also required for budding and insert into the protein-proximal leaflet, increasing lipid ordering and membrane thickness. Therefore, we hypothesize that the NEC-mediated membrane budding is driven by a mechanism that combines scaffolding with lipid ordering and lateral headgroup compression. Furthermore, we show that the MPRs can generate negative Gaussian curvature required for the formation and scission of the bud neck, which is consistent with the NEC-induced scission observed *in vitro* (21). Thus, the NEC is a self-contained membrane-budding machine capable of completing multiple actions in the budding process, at least *in vitro*.

### Electrostatic forces govern NEC-membrane interactions.

Previously, we showed that the NEC MPRs are necessary for the membrane recruitment of the soluble NEC *in vitro* through electrostatic interactions (21). Electrostatic interactions between basic residues and acidic lipids commonly serve to recruit cytoplasmic proteins to membranes (37), but the NEC is anchored in the INM through the TM helix of UL34 (30), which left uncertain the role of the MPRs in membrane budding. Here, we found that the MPRs, especially the UL31 MPR, are necessary for membrane budding and can induce lipid ordering. Both phenomena require basic clusters within the UL31 MPR. Basic clusters govern membrane interactions of proteins such as Src kinase (83), myristoylated alanine-rich C-kinase substrate (MARCKS) (84), neuromodulin (84), the BAR domain proteins (37, 85, 86), and HIV Gag (87). Moreover, it has been proposed that interactions of basic clusters with the membrane could promote negative membrane curvature by concentrating negatively charged lipids within the membrane (88). We hypothesize that interactions between the basic clusters within the UL31 MPR and the membrane drive formation of negative membrane curvature by the HSV-1 NEC. Considering that basic clusters are found in the MPRs of many UL31 homologs (Fig. 1G), their involvement in curvature formation may be a conserved feature of the NEC budding mechanism across different herpesviruses.

In addition to basic residues, the HSV-1 UL31 MPR contains six serines that are phosphorylated by the US3 viral kinase (31–33). The role of UL31 phosphorylation in nuclear egress is unclear but is thought to inhibit nuclear egress, because phosphomimicking serine-to-glutamate mutations of these six serines inhibits nuclear egress and HSV-1 replication (33), albeit by an unknown mechanism. In contrast, serine-to-alanine mutations of the same six serines in UL31, which mimic an unphosphorylated state, cause the accumulation of the perinuclear enveloped virions, i.e., budded capsids, in the perinuclear space (33). This phenotype is also observed when the US3 kinase is either missing or catalytically inactive (33, 39, 41, 42). Perinuclear enveloped virions may accumulate whenever UL31 cannot be phosphorylated either because phosphorylation facilitates de-envelopment (33) or due to excessive budding in the absence of inhibition (5).

Here, we observed that serine-to-glutamate mutations within the UL31 MPR blocked NEC-mediated budding *in vitro*. Glutamates, just like phosphates, are negatively charged, and since NEC-membrane interactions require a sufficiently high net positive charge of the UL31 MPR, introducing negative charges would disrupt proper NEC-membrane interactions. Indeed, phosphorylation and phosphomimicking mutations decrease protein-membrane interactions of the F-BAR domain of syndapin I (89), MARCKS (84, 90), neuromodulin (84), Cdc15 (91), PTEN (92), and dynamin I (93). Therefore, we hypothesize that phosphomimicking mutations block capsid nuclear egress by reducing the net positive charge of the UL31 MPR, thereby inhibiting NEC-mediated budding. Phosphorylation also introduces negative charges and would have a similar inhibitory effect on budding. We speculate that HSV-1 uses phosphorylation to inhibit the membrane-budding activity of the NEC and, thus, nuclear egress, by fine-tuning its membrane interactions. In this way, phosphorylation could serve as an off switch that prevents unproductive membrane budding prior to the arrival of the capsid. The need for a phosphatase to balance the action of the US3 kinase has been postulated, but the evidence has been lacking. Recently, however, HSV-1 UL21 was shown to bind the cellular protein phosphatase 1, directly causing dephosphorylation of UL31 *in vivo* (94). Since UL21 localizes to the nucleus and is involved in nuclear egress (95), by functioning as a phosphatase adaptor, UL21 could act as an on switch for NEC budding activity.

### Lipid ordering by NEC MPRs in combination with scaffolding generates negative mean curvature for the growing bud.

NEC-mediated membrane budding proceeds through two distinct steps: formation of the bud and scission of the bud neck. Bud formation requires generation of negative mean membrane curvature. The two most common mechanisms of curvature generation, be it positive or negative, are peripheral insertion of protein into lipid bilayers and scaffolding of the curvature by protein oligomers (reviewed in references 66 and 96–98). We propose that NEC-mediated membrane budding is driven by a mechanism that combines scaffolding with lipid ordering accompanied by lateral headgroup compression. Previous studies already established that the NEC oligomerizes into hexagonal scaffold-like coats on the inner surface of budded vesicles (21, 25, 26), and this oligomerization is essential for budding both *in vivo* and *in vitro* (21, 27, 28). Therefore, formation of negative membrane curvature by the NEC requires membrane scaffolding by NEC oligomers. Here, we demonstrated that highly basic MPRs of the NEC are also required for budding and can induce ordering of lipid headgroups and upper acyl chain regions in the protein-proximal leaflet of the membrane bilayer.

**FIG 9:**
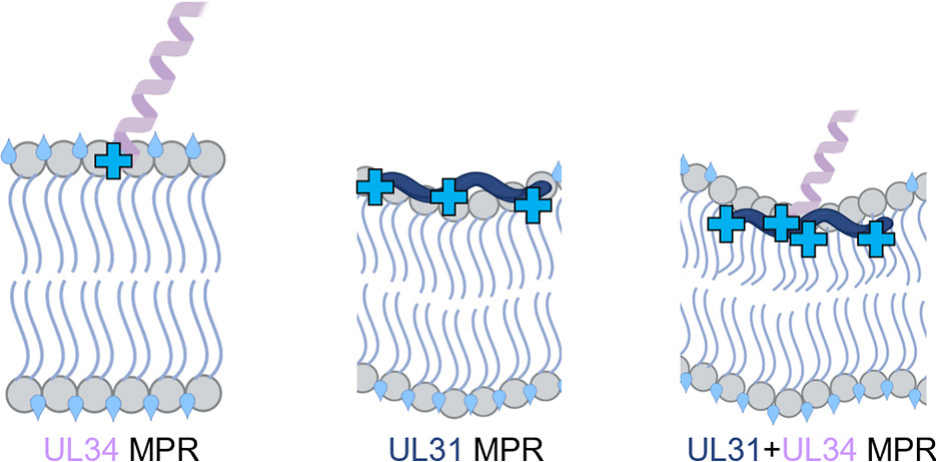
Model of negative mean membrane curvature generation by NEC MPR peptide-membrane interactions. The UL34 MPR peptide (light purple) with a C-terminal patch of basic residues (light blue cross) alone is insufficient to drive ordering of lipid headgroups and acyl chain region or displace water (light blue tear drops). The UL31 MPR peptide (dark blue) alone can induce ordering of the lipid headgroups, accompanied by outer leaflet dehydration. Combination of the UL31 and UL34 MPR peptides results in both lipid headgroup and upper acyl chain ordering along with membrane dehydration, resulting in the generation of local negative mean curvature in the protein-proximal leaflet. All images created with BioRender.com.

Our peptide studies suggest that lipid ordering is mediated by the unstructured UL31 MPR that engages membranes directly by inserting into the lipid headgroups. This peripheral membrane insertion can result in membrane dehydration leading to compression of the protein-proximal headgroups, ultimately generating local negative curvature (48). While many peripheral membrane proteins use amphipathic helices for membrane interactions, the UL31 MPR maintains a random-coil conformation even in the presence of membranes. Therefore, we think that the basic clusters within the UL31 MPR form fingertip-like projections that interact with the lipid headgroups in a multidentate manner (Fig. 9), similar to the membrane-interacting fusion loops (FLs) of class II viral fusogens, in which three or six FLs ensure sufficient grip on the membrane (99). It is unclear how many residues in the UL31 MPR insert into the membrane; however, given the low volume of protein detected in the membrane by NR, relatively few residues are involved. Ordering of the upper acyl chains could be due to the insertion of the UL31 MPR into the upper acyl chain region. Alternatively, the UL31 MPR could be drawing the headgroups together, compressing the protein-proximal leaflet, and constraining the motion of the upper acyl chains and, thus, the spin label located there without directly occupying the upper acyl chain region.

It has been proposed that protein-mediated ordering of the lipid headgroups results in dehydration of the protein-proximal leaflet leading to tighter lipid packing and shrinking of the local area (48), leading to the formation of negative mean membrane curvature. On a flat substrate, this would result in membrane thickening, and, indeed, the NR experiments revealed a thickening of the tethered bilayer after incubation with NEC220. Therefore, we hypothesize that the MPR-induced ordering of lipid headgroups and upper acyl chains generates negative mean membrane curvature. Given that MPR-membrane interactions would generate curvature only locally, we hypothesize that generation of negative mean membrane curvature over a large membrane area requires NEC oligomerization into a hexagonal scaffold. As the membrane-tethered NEC heterodimers oligomerize into the hexagonal scaffold, they can create compressive pressures that would generate negative mean curvature, driving vesicle budding (100, 101). In this manner, the lipid ordering and oligomerization work together to mold the associated membrane into a spherical shape.

The cooperative effect of the UL31 and UL34 MPR peptides on lipid ordering as well as a greater depth of insertion of UL31 MPR peptides in the presence of UL34 MPR peptide suggest that the UL31 and the UL34 MPR peptides interact in the presence of the membrane, which we detected by both DEER and chemical cross-linking. Whereas the UL31 MPR interacts with the membrane directly, the UL34 MPR likely assists in positioning the UL31 MPR for optimal penetration necessary to induce the required degree of lipid ordering and, thus, headgroup compression (Fig. 9). The HSV-1 UL34 MPR is predicted to form an α helix and, indeed, becomes α-helical in the presence of the membrane. Although this region was unresolved in the HSV-1 NEC crystal structure (27), the corresponding region in HCMV (78, 79) and PRV (27, 80) homologs forms an α helix oriented perpendicularly to the membrane. To reflect this, we have modeled the UL34 MPR peptide such that its α-helical segment is oriented perpendicularly to the membrane, which positions its basic cluster to interact with the membrane and, presumably, with the UL31 MPR (Fig. 9). Although the local density of the MPR peptides on membranes in the ESR experiments may exceed the local density of MPRs in the context of the NEC, both NEC220 and NEC220Δ40 also cause lipid headgroup ordering. This suggests that the membrane remodeling properties of the MPR-derived peptides model the behavior of the NEC. Nonetheless, future studies should explore the lipid ordering by the NEC in more detail.

### NEC can achieve scission by generating negative Gaussian curvature.

In addition to generating membrane buds, the NEC can also drastically constrict the necks of the budded vesicles via membrane remodeling *in vitro* (21). We found that UL31 and UL34 MPR peptides can generate NGC, which is the type of curvature topologically required for formation of the scission neck, and that their effect on NGC formation is cooperative, which parallels their effect on lipid ordering observed by the ESR. Based on the quantitative measurements of NGC in the MPR-induced lipid phases, we estimate that the NEC could generate the membrane scission neck with an inner diameter of 7.2 nm, which is consistent with the diameters of the necks formed by mitochondrial fission proteins capable of spontaneous scission (64) and with the theoretical calculations (68). The ability of the MPRs to generate tight membrane curvatures found in scission necks in other biological systems suggest that they contribute likewise to NEC-induced bud scission observed *in vitro* (21, 22). We note, however, that the local density of the MPR peptides on membranes in the SAXS experiments may exceed the local density of MPRs in the context of the NEC. Future studies should investigate NGC generation by the NEC.

While the NEC demonstrates an intrinsic membrane scission ability *in vitro* (21, 22), efficient nuclear egress at least in some cell types (35) if not in others (34) requires ESCRT-III machinery. Several enveloped viruses, notably HIV, recruit cellular endosomal sorting complexes required for transport III (ESCRT-III) (reviewed in references 102–106) to mediate scission during viral budding. ESCRT-III proteins accomplish scission of the bud neck by forming a spiral polymer on the inward face of the neck and constricting it (107, 108). Not all enveloped viruses, however, recruit ESCRT-III proteins for membrane scission. For example, influenza A virus deploys the amphipathic helix within its M2 channel (109) (reviewed in references 110 and 111), which has been proposed to mediate neck scission through a mechanism that involves the generation of the NGC (63).

The neck generated by the NEC may not be sufficiently narrow to trigger spontaneous membrane scission with high enough efficiency required for vesicle release in the context of HSV-1 nuclear budding. If so, low efficiency of this NEC-mediated scission could, in principle, account for the need to recruit ESCRT-III machinery to increase the efficiency of membrane bud scission during nuclear egress in a cell type-specific manner. This scenario is reminiscent of Ebola virus, where the viral matrix protein VP40 mediates membrane budding *in vitro* (112) yet recruits ESCRT-III machinery *in vivo* (113, 114) (reviewed in reference 115). Future experiments will address the coordination of efforts between the NEC and the ESCRT-III proteins in mediating nuclear egress.

### A model of membrane curvature generation by the NEC.

While the NEC can form both negative mean curvature and NGC, it is unknown what determines the transition from dome formation to neck formation and scission. We postulate that this switch depends on the oligomeric state of the NEC. Within the hexagonal lattice, the NEC heterodimers adopt vertical orientations (21, 25), yet the NR measurements suggest that a significant fraction of the NEC may have tilted or flat orientations. Therefore, we hypothesize that on the membrane, there are regions with high and low densities of NECs. At higher NEC densities, oligomerization would promote an upright orientation, whereas at lower NEC densities, individual NEC heterodimers would experience greater orientational freedom.

**FIG 10:**
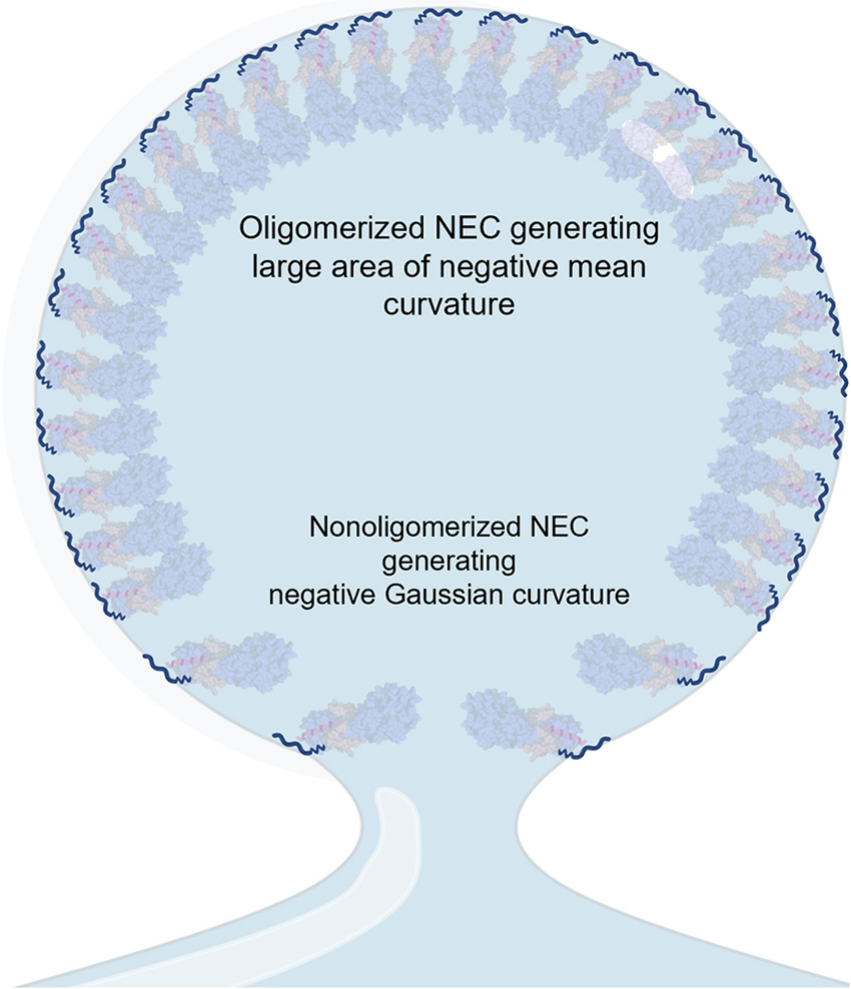
Model of negative mean membrane curvature and negative Gaussian curvature generation by NEC MPR peptide-membrane interactions. Oligomerized NEC forces MPRs to work in concert and generate larger areas of negative mean curvature. Nonoligomerized NEC adopts a more flexible orientation and the MPRs generate negative Gaussian curvature to perform scission. All images created with BioRender.com.

Putting together our experimental observations, we propose the following model of curvature generation by the NEC (Fig. 10). We hypothesize that in areas with high NEC density, such as the body of the budding vesicle, the NEC oligomerizes into the hexagonal scaffold. While the MPRs of the NECs have the capacity to generate NGC, in the body of the bud, the hexagonal scaffold forms a rigid frame that constrains the membrane into a defined spherical architecture, promoting negative mean curvature. As more NECs are recruited and oligomerize, the hexagonal scaffold expands and the budding vesicle grows. However, at membrane regions not covered by the hexagonal coat, such as near the rim of the bud, NECs may mainly exist as unconstrained individual heterodimers or, perhaps, individual hexamers. At these regions, membrane interactions by individual NEC heterodimers could facilitate the induction and stabilization of NGC to produce saddle-shaped deformations necessary for scission (Fig. 10).

Our experiments do not directly address the behavior of an intact NEC anchored in the membrane by the TM of UL34 (residues 248 to 272). However, we hypothesize that the TM is unlikely to constrain MPR-membrane interactions in a significant way because UL34 residues 204 to 234 are predicted to be unstructured and could accommodate the optimal MPR conformation in the nonoligomerized state. Moreover, the UL34 TM is dispensable for budding *in vitro* (21) and can be substituted for a heterologous TM *in vivo* (30). Nonetheless, future experiments should address how membrane interactions by the MPRs in the context of the full NEC are coordinated with NEC oligomerization to bring about membrane budding.

## MATERIALS AND METHODS

### Cloning of expression constructs.

Cloning of constructs encoding HSV-1 strain F UL31 with boundaries 1 to 306, 41 to 306, and 51 to 306 is described elsewhere (21). Primers used for cloning are listed in Table S3 in the supplemental material. Site-directed mutagenesis of the UL34 mutant with boundaries 1 to 185 was performed by restriction digest and ligation with SalI and NotI. Site-directed mutagenesis of the UL31 mutant with boundaries 1 to 306 (S11E/S24E/S43E) was performed by two rounds of inverse PCR on a full-length UL31 that already contained an S43E mutation and blunt-end ligation. S11E/S24E/S26E/S27E/S40E/S43E was generated by three sequential inverse PCR and blunt-end ligation reactions. The first round was to generate S11E followed by blunt-end ligation and inverse PCR to generate S11E/S24E/S26E/S27E, followed by blunt-end ligation and inverse PCR to generate S11E/S24E/S26E/S27E/S40E/S43E. Site-directed mutagenesis of UL31 mutants with boundaries 41 to 306 (R49S/K50S, R41S/K42S/R49S/K50S, L44A, S43E, R41S/K42S/S43R/L44K/R49S/K50S, R41S/K42S/P45R/P46K/R49S/K50S, R41S/K42S/H47R/A48K/R49S/K50S, P45A/P46A, ^41^KSPKLHRARP^50^) was performed by inverse PCR. Site-directed mutagenesis for the UL31 mutant with boundaries 41 to 306 and mutations R41S/K42S was performed by restriction digest and ligation with BamHI and NotI. Site-directed mutagenesis of UL31 mutant with boundaries 47 to 306 containing mutations H47R/A48K was performed by inverse PCR.

10.1128/mBio.01548-21.10 TABLE S3 List of primers used for cloning described in Materials and Methods. All primers are listed in the 5′–3′ direction. Mutations are bolded, and restriction digest sites are underlined and listed underneath applicable primers. Download Table S3, PDF file, 0.1 MB. Copyright © 2021 Thorsen et al. 2021 Thorsen et al. https://creativecommons.org/licenses/by/4.0/ This content is distributed under the terms of the Creative Commons Attribution 4.0 International license.

A gene block with the codon-optimized DNA sequence for UL31 residues 1 to 50 was purchased from Integrated DNA Technologies (IDT). PCR was performed on the gene block with primers containing the restriction digest sites for BamHI and NotI. The resulting PCR product was purified and digested with BamHI and NotI and ligated into pGEX-6P-1 with an N-terminal glutathione *S*-transferase (GST) tag for solubility and affinity purification purposes. Inverse PCR followed by blunt end ligation was used to develop UL31^(C1–50)^ and UL31^(1–C51)^. Three rounds of inverse PCR and blunt end ligation were needed for UL31^(C1–50)^ due to introduction of point mutations.

All constructs generated in this work are listed in Table S3.

### Protein purification.

Plasmids encoding HSV-1 UL31 and UL34 were cotransformed into Escherichia coli LOBSTR-BL21(DE3) cells and expressed at 25°C for 16 h after lactose-derived autoinduction (116). Cells were resuspended in lysis buffer [50 mM HEPES, pH 7.5, 500 mM NaCl, 0.5 mM Tris(2-carboxyethyl)phosphine hydrochloride (TCEP), 10% glycerol] in the presence of Complete protease inhibitor (Sigma-Aldrich) and lysed using a M-110S microfluidizer (Microfluidics). Cell lysate was spun down at 12,500 rpm in a Beckman J2-21 floor centrifuge. All purification steps were performed at 4°C. The clarified cell lysate was first passed over Ni-NTA Sepharose resin (GE Healthcare). The resin was washed with wash buffer (lysis buffer containing 20 to 40 mM imidazole). Bound protein was eluted with elution buffer (lysis buffer containing 250 mM imidazole) and loaded onto glutathione Sepharose resin (GE Healthcare) to separate bound NEC from excess His_6_-SUMO-UL31. After washing with lysis buffer containing 1 mM EDTA, His_6_-SUMO and GST tags were cleaved on the glutathione Sepharose column for 16 h using PreScission protease produced in-house using a GST-PreScission fusion protein expression plasmid. Cleaved NEC and His_6_-SUMO were eluted from the glutathione Sepharose column with lysis buffer and diluted to 100 mM NaCl with 50 mM HEPES, pH 7.5, 0.5 mM TCEP, 10% glycerol. NEC was separated from His_6_-SUMO using a cation exchange resin (HiTrap SP XL; GE Healthcare) with a 200 mM to 600 mM NaCl gradient in 20 mM HEPES, pH 7.0, 0.5 mM TCEP. Each NEC construct was purified to homogeneity as assessed by 12% SDS-PAGE and Coomassie staining. Fractions containing NEC were diluted to 100 mM NaCl with 20 mM HEPES, pH 7.0, 0.5 mM TCEP and concentrated up to ∼1.5 mg/ml and stored at −80°C to avoid aggregation and degradation at 4°C. Protein concentration was determined by absorbance measurements at 280 nm. The typical yield was 0.5 mg per liter TB culture. UL31 1 to 50 peptides UL31^(1–50)^, UL31^(C1–50)^, and UL31^(1–C51)^ were expressed and purified with the same buffers. Briefly, these three UL31 MPR peptides were passed over glutathione Sepharose resin and washed with lysis buffer containing 1 mM EDTA. The GST tag was cleaved as outlined above. The glutathione Sepharose column eluate was then concentrated to 500 μl and passed over an S75 10/300 size exclusion column (GE Healthcare) with 20 mM HEPES, pH 7.0, 100 mM NaCl, 0.5 mM TCEP. Fractions containing UL31 MPR peptides were concentrated up to 5 mg/ml and stored at −80°C.

### Liposome preparation.

Liposomes were prepared as described previously (21). Briefly, MLVs were made by mixing 1-palmitoyl-2-oleoyl-sn-glycero-3-phosphocholine (POPC), 1-palmitoyl-2-oleoyl-sn-glycero-3-phospho-l-serine (POPS), and 1-palmitoyl-2-oleoyl-sn-glycero-3-phosphate (POPA) (Avanti Polar Lipids) at a molar ratio of 3:1:1 POPC/POPS/POPA, followed by vacuum drying the mixture and resuspending in 200 μl 20 mM HEPES, pH 7.0, 100 mM NaCl, 0.5 mM TCEP to shake for 0.5 h in a 37°C incubator (21, 117). The lipid mixture was then vortexed and used immediately. For GUVs, lipids were mixed at a molar ratio of 58% POPC–11% POPE–9% POPA–9% POPS–5% cholesterol–5% DGS-NTA–3% POPE Atto594, of which 5 μl was spread on the surface of an ITO-covered slide and vacuum desiccated for 30 min. A vacuum-greased O-ring was placed around the dried lipid mixture, and the VesiclePrep Pro (Nanion Technologies) was used to produce an AC field (sinusoidal wave function with a frequency of 8 Hz and amplitude 2V) before adding 270 μl of lipid swelling buffer (300 mM sucrose dissolved in 5 mM Na-HEPES, pH 7.5). A second ITO-covered slide was used to cover the lipid/buffer mixture after 3 min, followed by a 2-h swell and a 5-min fall step. GUVs were used immediately and diluted 1/20 with 20 mM HEPES, pH 7.0, 100 mM NaCl, 0.5 mM TCEP.

### Membrane cosedimentation assay.

Three micrograms of protein was incubated with or without 15 μg freshly prepared MLVs (as detailed above) at 20°C for 30 min. The samples were centrifuged at 16,000 × *g* for 20 min at 4°C. Aliquots of protein-MLV pellet and protein supernatant were analyzed by 12% SDS-PAGE and Coomassie staining. The amount of protein that pelleted with MLVs was determined by densitometry analysis of gels imaged using a LI-COR Odyssey CLx imager and quantified using ImageJ. For each protein, band intensities of the pelleted protein were integrated and expressed as a percentage of the total integrated intensity of protein bands in the pelleted sample and supernatant sample. Background levels of pelleted protein in the absence of vesicles were subtracted from levels of protein sedimentation in the presence of vesicles. Each experiment was done with duplicate technical replicates with at least three biological replicates, and the average value and standard error of the mean is reported. Data were plotted using GraphPad Prism 9.0.

### GUV budding assay.

Fluorescently labeled GUVs were coincubated for 3 min with the soluble NEC and the membrane-impermeable dye Cascade Blue hydrazide (ThermoFisher Scientific). The GUV contained 18% negatively charged lipids (58% POPC–11% POPE–9% POPA–9% POPS–5% cholesterol–5% DGS-NTA–3% POPE Atto594), which closely resembles the inner nuclear membrane of uninfected cells (118, 119). The composition of the nuclear membrane in HSV-1-infected cells is unknown but may be different from that of uninfected cells. Budding events manifested as the appearance of intraluminal vesicles (ILVs) containing Cascade Blue within the GUVs (Fig. 1C). Five microliters of the above-described GUV composition and 2 μl Cascade Blue hydrazide were mixed with a final concentration of 1.5 μM NEC for a total volume of 100 μl. Each sample was visualized using a Nikon A1R confocal microscope. Background levels of intraluminal vesicles were counted in the absence of NEC and subtracted from counts of intraluminal vesicles in the presence of NEC. Experiments were performed with at least 3 technical replicates and at least 3 biological replicates. All counts were normalized to NEC220-His_8_ budding. Levels of budding are broken down into three categories based on statistical significance, poor (0 to 49%, ***, *P* < 0.0005), moderate (50 to 74%, *, *P* < 0.05; **, *P* < 0.005), and efficient (75 to 100%). The NEC220Δ50-RKRK-His_8_ and NEC220Δ40-P45A/P46A-His_8_ mutants were exceptions because they supported efficient budding (85% and 75%, respectively), yet the *P* value was <0.05. The standard error of the mean is reported from at least three individual experiments. Data were plotted using GraphPad Prism 9.0.

### Coflotation assay.

NEC sensitivity to membrane curvature was tested using coflotation as described previously (120). Briefly, 1.5 μg NEC was incubated with or without large unilamellar vesicles (LUVs) (POPC, POPS, and POPA mixed in a 3:1:1 molar ratio as previously described [21]) at room temperature for 20 min in 50 ml phosphate-buffered saline (PBS). KCl was added to 200 mM concentration to reduce nonspecific protein-membrane interactions, and samples were incubated for 15 min at room temperature. OptiPrep (Sigma) was added to a final concentration of 30% in a 500-ml volume. Samples were placed at the bottom of a 5-ml centrifugation tube (Beckmann) and overlaid with 4 ml 15% OptiPrep and 500 ml 3% OptiPrep in PBS. The samples were next centrifuged in a Beckman SW-55 Ti rotor at 246,000 × *g* for 3 h at 4°C, and 1-ml fractions were collected beginning at the top. Protein was precipitated with 20% trichloroacetic acid for 30 min on ice. Sample was washed with 750 μl cold acetone and then spun in a tabletop centrifuge for 10 min at 14,000 rpm. This was repeated for a total of 3 washes. Samples were then analyzed by Western blotting for UL31 as previously described (21). The standard error of the mean is reported from at least two individual experiments. Data were plotted using GraphPad Prism 9.0.

### Neutron reflectometry.

Silicon wafers (100; n-doped to a conductivity of 1 to 100 Ω cm) of 5-mm thickness and 75-mm diameter were coated with 40 Å Cr for adhesion purposes, followed by 140 Å Au by magnetron sputtering on a Denton Vacuum Discovery 550 sputtering system at the National Institute for Standards and Technology (NIST) Center for Nanoscale Science and Technology cleanroom. The substrate was then immersed for 8 h in an ethanolic solution of the thiol-lipid linking molecule HC18 [(Z20-(Z-octadec-9-enyloxy)-3,6,9,12,15,18,22-heptaoxatetracont-31-ene-1-thiol)] (121) and βME (β-mercaptoethanol) in a 3:7 molar ratio and a total concentration of 0.2 mM. The resulting self-assembled monolayer (SAM) was rinsed in ethanol and dried in a nitrogen stream. The coated surface of the sample wafer was mounted facing a 100-μm reservoir defined by a 65-mm inner diameter cylindrical Viton gasket separating the sample wafer from a rough backing wafer (122). The backing wafer was perforated by single inlets and outlets, which were coupled by IDEX Health and Science (Oak Harbor, WA) flat-bottomed fittings to external tubing for solution exchanges, which were performed using at least 7.5 ml flowing at 2.5 ml/min. To prepare multilamellar vesicles (MLVs), a solution of POPC/POPS/POPA in a 3:1:1 molar ratio was prepared at 10 mg/ml in 1 M NaCl, subjected to 40 min of bath sonication, and injected into the sample cell. Incubation proceeded for at least 1.5 h, followed by flushing with pure water to lyse the vesicles via osmotic stress, forming a sparsely tethered lipid bilayer membrane.

NR experiments were carried out on the MAGIK vertical reflectometer (123) at the NIST Center for Neutron Research (NCNR). A monochromatic beam of wavelength λ = 5.000 Å impinged on the interface between the coated surface of the sample wafer and the liquid in the sample cell reservoir. The presample collimating slits were chosen to maintain a constant illuminated interface area for each measured angle θ. The postsample collimation was chosen to allow the entire reflected beam to impinge on the detector, which was positioned at angle 2θ relative to the incoming beam direction to measure specular reflection. Each reflectivity curve covered a range in scattering wavevector *Q* = 4πλ^−1^sin(θ) from 0.008 Å^−1^ to 0.251 Å^−1^.

The reflectivity was calculated as *R*(*Q*) = [*I*(*Q*)-*I_B_*(*Q*)]/*I*_0_(*Q*). Here, *I*(*Q*) is the measured count rate (normalized to a much larger monitor count rate to account for fluctuations in beam intensity) at the specular condition. *I_B_*(*Q*) is the background intensity, which arises primarily from incoherent scattering from the liquid reservoir and is calculated by linear interpretation of the intensities measured with the detector at off-specular positions 1.5θ and 2.5θ. *I*_0_(*Q*) is the incident beam intensity and is directly measured through the silicon substrate at θ = 0 with the detector positioned in line with the incident beam.

NR data were analyzed using the composition space modeling procedures described previously (124). Briefly, the composition space model arranges the known molecular components of the tethered bilayer and protein at the substrate surface; any unfilled space is assumed to be filled with water. Because the neutron scattering length density (nSLD) of each component is known or can be estimated from its elemental composition and molecular volume, an average nSLD profile can be calculated as a function of distance from the substrate surface. This nSLD profile in turn corresponds to a predicted *R*(*Q*) that can be optimized to the experimental data, using as parameters the spatial arrangement of the molecular components. Replacing all H_2_O in the membrane-bathing buffer with D_2_O provides contrast and allows unambiguous determination of the nSLD profile associated with both measured *R*(*Q*) curves by simultaneous optimization of the two contrast conditions (125).

The protein profile was parameterized in two ways for comparison. The Catmull-Rom spline, or “freeform” profile, makes no assumptions about the shape of the volume occupied by the protein but does assume that the nSLD of the protein is equal to its average value for the entire protein. Alternatively, an “orientation” profile is used, in which the protein profile is calculated from the crystallographic structure of the NEC complex (PDB entry 4ZXS) (27) rotated by Euler angles α and β, with the volume of the MPRs represented in the appropriate molar ratio by a smoothed box function. The Euler angles are defined in an *x-y-z* extrinsic rotation scheme, where the *z* axis is codirectional with the surface normal. Because NR is sensitive to the nSLD only in the *z* direction, it is not sensitive to the final rotation about the *z* axis, γ. Each profile was convolved with a width 4.1-Å Gaussian function to account for surface roughness. The orientation models require fewer parameters than freeform models and do account for spatial variations in nSLD but assumes a single, rigid structure for the protein.

Optimization was performed on the Bridges (126, 127) high-performance computing system using the DREAM Markov chain Monte Carlo (MCMC) algorithm (128) implemented in the software package Refl1D (129). Confidence intervals (CI) on parameters and model predictions were calculated from parameter distributions derived from 14.4 million DREAM samples after the optimizer had reached steady state.

### Lipid preparation for electron spin resonance.

POPC, POPS, and POPA were mixed at a 3:1:1 molar ratio with 0.5% (mol/mol) spin-labeled lipid in chloroform and dried under N_2_ gas. The dried mixture was placed under vacuum overnight to remove any remaining chloroform. To prepare SUVs, dried lipids were resuspended in pH 7.0 buffer (20 mM HEPES, 100 mM NaCl, 0.5 mM TCEP) and sonicated in an ice bath for at least 20 min or until the solution became clear. The SUV solution was then subject to ultracentrifugation at 13,000 rpm for 10 min for further clarification to remove the possible membrane debris.

### UL31 and UL34 MPR peptides.

Peptides UL31^(41–50)^, UL31^(C40–50)^, UL31^(41–C51)^, UL31^scr(41–C51)^, UL31^(C40–50 R41S/K42S)^, UL31^(22–42)^, UL31^(C21–42)^, UL31^(22–C43)^, UL31^scr(22–C43)^, UL34^(174–194)^, and UL34^scr(174–194)^ were purchased from Peptide 2.0. All peptides were N-terminally acetylated, C-terminally amidated, and ≥95% pure.

### Peptide labeling.

For peptide labeling, desired amounts of UL31 or UL34 peptides were dissolved in pH 8.0 buffer (5 mM HEPES, 10 mM MES, 150 mM NaCl) and mixed with 10-fold excess MTSL [S-(2,2,5,5-tetramethyl-2,5-dihydro-1H-pyrrol-3-yl) methyl methanesulfonothioate] dissolved in ethanol (200 mM); the volume of the ethanol added was less than 5% of the total volume. The mixtures were kept overnight in the dark at room temperature (RT) as previously described (50). The spin-labeled peptides were then subjected to purification using fast performance liquid chromatography with a GE Superdex peptide 10/300 GL at a flow rate of 0.2 ml/min for 150 min. The fractions containing the peptides were lyophilized overnight and dissolved in pH 7.0 buffer (20 mM HEPES, 100 mM NaCl, 0.5 mM TCEP).

### CW-ESR on lipid probes.

The desired amount of peptide and SUVs (3:1:1 POPC/POPS/POPA molar ratio) were mixed at RT for 30 min. The final amount of the lipid in each sample was 1 mg. The ESR spectra were collected on an ELEXSYS ESR spectrometer (Bruker Instruments, Billerica, MA) at X-band (9.5 GHz) at 25°C using an N_2_ temperature controller (Bruker Instruments, Billerica, MA). The ESR spectra from the labeled lipids were first denoised whenever necessary (130). They were then analyzed using the NLLS fitting program based on the stochastic Liouville equation (131, 132) using the microscopic order macroscopic disorder (MOMD) model as in previous studies (47–49, 133, 134). The *A* and *g* values of the spins are determined using the low-temperature ESR measurements. Two sets of parameters that characterize the rotational diffusion of the nitroxide radical moiety in spin labels are generated. The first set consists of *R*_⊥_ and *R*‖, which are the rates of rotation of the nitroxide moiety around a molecular axis perpendicular and parallel to the preferential orienting axis of the acyl chain. The second set consists of the ordering tensor parameters, *S*_0_ and *S*_2_, which are defined as *S*_0_ = <*D*_2,00_> = <1/2(3cos^2^θ − 1)> and *S*_2_ = <*D*_2,02_ + *D*_2,0-2_> = <√(3/2)sin^2^θcos2φ>, where *D*_2,00_, *D*_2,02_, and *D*_2,0-2_ are the Wigner rotation matrix elements and θ and φ are the polar and azimuthal angles for the orientation of the rotating axes of the nitroxide bonded to the lipid relative to the director of the bilayer, i.e., the preferential orientation of lipid molecules (48, 132), with the angular brackets implying ensemble averaging.

*S*_0_ indicates the strength of the alignment of the chain segment to which the nitroxide is attached along the normal to the lipid bilayer, which is correlated with hydration/dehydration of the lipid bilayers (47). *S*_2_ is the measurement of the molecular nonaxiality of the motion of the spin label. It was found to be much smaller than *S*_0_, with much less sensitivity to changes in bilayer structure in our studies. Therefore, *S*_0_ is the more important parameter for this study. The estimated error of *S*_0_ from the NLLS fit for the spectra (the typical standard deviation obtained in the fitting) is about ±0.005 to 0.008 from at least three individual experiments.

### Vesicle sedimentation assay for partition ratio.

Sucrose-loaded LUVs (3:1:1 POPC/POPS/POPA) were prepared as described previously (135, 136). Approximately 100 μM peptide was incubated with 10 mM LUVs in a 1:1 ratio for 1 h at 37°C. The final lipid concentration was confirmed by a phosphate assay (137). The mixtures were then centrifuged at 100,000 × *g* for 1 h at 25°C. The pellets were washed briefly before ESR measurement. The amount of the spin-labeled peptides in the supernatant and the pellets were determined by CW-ESR using the build in double integration tool in the Bruker XEPR program. The partition ratio for peptide is defined as the amount of peptide in the pellet to the amount of peptide in the supernatant. Data shown are from three independent experiments, and the standard deviation is reported.

### Power saturation CW-ESR.

The spin-labeled peptides were mixed with liposomes, and power saturation ESR spectra were collected in the presence of argon, O_2_, or NiEDDA. O_2_ and NiEDDA are spin relaxation reagents. Their concentration around the spins is correlated with their collision with the spins and, thus, affects the power saturation curve of the spins (i.e., peak-to-peak amplitude versus microwave power), from which the accessibility parameters Π(O_2_) and Π(NiEDDA) are calculated (138, 139). The CW-ESR measurement spectra were collected on an ELEXSYS ESR spectrometer at X-band (9.5 GHz) at RT. The power saturation experiments were performed in air, argon, and 20 mM Ni(II)-diammine-2,2’-(ethane-1,2-diyldiimino) diacetic acid (NiEDDA) with argon conditions. The latter two conditions were achieved by repeatedly degassing and saturating the sample with argon (58). Under each condition, the spectra were recorded as a function of microwave power, which was varied from 0.1 mW to 200 mW in 30 steps. The number of scans depended on the quality of the signal. The half-saturation parameter (*P*_1/2_) is obtained by fitting the equation *A* = I*√P* [1 + (2^1/ε^ − 1)*, *P*/*P*_1/2_]^−ε^, where *P* is the microwave power applied, *A* is the peak-to-peak value of the central line of the spectra, and ε is the line-homogeneity parameter that we obtained from the fitting (usually ε = 1.5 formed the best fit). The accessibility parameter Π(O_2_) and Π(Ni) are calculated by the equations Π(O_2_) = [*P*_1/2_(O_2_)/Δ*H*(O_2_) − *P*_1/2_(Ar)/Δ*H*(Ar)]/[*P*_1/2_(ref)/Δ*H*(ref)] and Π(Ni) = [*P*_1/2_(Ni)/Δ*H*(Ni) − *P*_1/2_(Ar)/Δ*H*(Ar)]/[*P*_1/2_(Ref)/Δ*H*(Ref)], where Δ*H* is the line width of the central line measured at 2 mW. The insertion depth parameter Φ, which is independent of the reference, was calculated by the equation Φ = ln[Π(O_2_)/(Π(NiEDDA)] (58, 140). *P*_1/2_(ref) and Δ*H*(ref) are typically obtained from a standard sample, 2,2-diphenyl-1-picrylhydrazyl (DPPH), to account for differences in resonator efficiencies (*P*_1/2_) and compensates for differences in the spin-spin relaxation time (*T*_2_) by factoring in the central line width (Δ*H*) (141). However, the [*P*_1/2_(ref)/Δ*H*(ref)] term has been cancelled in the calculation of the insert depth parameter Φ. Therefore, neither *P*_1/2_(ref) nor Δ*H*(ref) was used in calculations. All experiments were done at least in duplicate to ensure reproducibility. Error reported is the standard deviation.

### SAXS.

Lyophilized phospholipids 1,2-dioleoyl-*sn*-glycero-3-phospho-l-serine (sodium salt) (DOPS) and 1,2-dioleoyl-*sn*-glycero-3-phosphoethanolamine (DOPE) were purchased from Avanti Polar Lipids and dissolved in chloroform at 20 mg/ml to produce individual lipid stock solutions. The lipid stock solutions were mixed at a molar ratio of 1:4 DOPS/DOPE, evaporated under nitrogen, and desiccated overnight under vacuum to form a dry lipid film. The lipid film was resuspended in aqueous pH 7.4 buffer (10 mM HEPES, 140 mM NaCl) to a concentration of 20 mg/ml. The resulting lipid suspension was incubated overnight at 37°C, sonicated until clear, and extruded through a 0.2-μm-pore-size Anotop syringe filter (Whatman) to form SUVs.

Lyophilized peptides UL31^(41–50)^, UL31^(22–42)^, and UL34^(174–194)^ were solubilized in aqueous pH 7.4 buffer (10 mM HEPES, 140 mM NaCl) and mixed with SUVs at peptide-to-lipid charge ratios (ch P/L) of 1:4, 1:2, 1:1, 3:2, and 2:1, which correspond to peptide-to-lipid molar ratios (mol P/L) of 1:140, 1:70, 1:35, 3:70, and 2:35 for UL31^(22–42)^ and UL34^(174–194)^, and 1:80, 1:40, 1:20, 3:40, and 1:10 for UL31^(41–50)^. Peptide-lipid samples were hermetically sealed into quartz capillaries (Mark-tubes, no. 4017515; Hilgenberg GmbH) and incubated at 37°C. SAXS measurements taken at the Stanford Synchrotron Radiation Lightsource (SSRL; beamline 4-2) using monochromatic X-rays with an energy of 9 keV. The scattered radiation was collected using a DECTRIS PILATUS3 × 1M detector (172-μm pixel size) and the resulting two-dimensional SAXS powder patterns integrated using the Nika 1.82 (142) package for Igor Pro 7.08 (WaveMetrics).

The integrated scattering intensity *I*(*Q*) versus *Q* was plotted using OriginPro 2017 and the ratios of the measured peak *Q* positions were compared with those of permitted reflections for different crystal phases to identify the phase(s) present in each sample. For a cubic phase, *Q* = (2π/*a*)√(*h*^2^ + *k*^2^ + *l*^2^), and for a hexagonal phase, *Q* = (4π/(*a*√3))√(*h*^2^ + *hk* + *k*^2^) were used, where *a* is the lattice parameter and *h*, *k*, and *l* are the Miller indices of the reflection. Linear regressions of measured *Q* versus √(*h*^2^ + *k*^2^ + *l*^2^) for cubic phases and measured *Q* versus √(*h*^2^ + *hk* + *k*^2^) for hexagonal phases were performed. The slope *m* of each regression was then used to calculate the respective cubic (*m *=* *2π/*a*) and hexagonal [*m *=* *4π/(*a*√3)] lattice parameters. For a lamellar phase, the periodicity, *d*, can be calculated from the relation of *Q *=* *2π*n*/*d*, where *n* is the order of the reflection.

For a cubic phase, the average Gaussian curvature per unit cell is calculated using the equation <*K*> = (2πχ)/(*A*_0_*a*^2^), where the Euler characteristic, χ, and the dimensionless surface area per unit cell, *A*_0_, are constants specific to each cubic phase (143). For *Pn3m*, χ = −2 and *A*_0_ = 1.919. For *Im3m*, χ = −4 and *A*_0_ = 2.345.

### DEER spectroscopy.

Approximately 50 μM peptide or peptide mixture were incubated with 10 mM SUVs (3:1:1 POPC/POPS/POPA molar ratio) in a 1:1 ratio for 10 min at 25°C. Deuterated glycine was added to reach a final concentration of 20% (wt/vol). The samples were transferred to an ESR tube and rapidly frozen in liquid nitrogen. Standard four-pulse DEER ESR experiments were performed using a Bruker 34-GHz Q-band ELEXSYS ESR spectrometer (Bruker Instruments, Billerica, MA) at 60 K. A pulse sequence with π/2 − π − π pulse widths of 16 ns, 32 ns, and 32 ns, respectively, and a 32-ns π pump pulse was routinely used or adjusted by the standard setup experiments. The frequency separation between detection and pump pulses was typically 70 MHz or else determined in standard setup experiments. Typical evolution times were 6 μs, with signal averaging from 8 to 10 h. The spectra were subject to wavelet denoising (130) as necessary. The background signals were removed from the raw time domain signals, and the distances were reconstructed from the baseline-subtracted signals using the singular value decomposition (SVD) method (73). The *P*(*r*) distributions obtained by this method were compared to the ones using the Tikhonov regulation method and refined by the maximum entropy method as previously described (69, 144). In our case, the differences between these two methods were not significant. The distance distribution is further fitted by a Gaussian distribution to obtain the position and width of the peak. The data were analyzed using Origin (OriginLab Inc.). Data reported are from at least two individual experiments with the error reported as the standard error of the mean.

### Chemical cross-linking.

A total of 50 μM peptide(s) in PBS was incubated with or without 1 mM SUVs (3:1:1 POPC/POPS/POPA molar ratio) (<100 nm) for 10 min at room temperature. In cross-linking experiments all peptides were N-terminally acetylated and C-terminally amidated. In the case of two peptide mixtures, 25 μM each peptide was used. SM(PEG)_6_ cross-linker (ThermoFisher Scientific), containing *N*-hydroxysuccinimide and maleimide groups that react with primary amines and sulfhydryls, respectively, was added at a 50-fold molar excess, and the samples were incubated for 30 min at room temperature. The reaction was stopped by adding Tris-HCl, pH 8.0, to a final concentration of 25 mM and glutathione to a final concentration of 50 mM. Samples were analyzed by 16% Tris-tricine–SDS-PAGE and Coomassie staining. For each sample, band intensities of the higher molecular weight cross-linked protein were integrated and expressed as a percentage of the integrated intensity of uncrosslinked protein. Each experiment was done in duplicate, and the average value and standard error of the mean are reported.

### CD.

Far-UV CD spectra of peptides with or without SUVs were recorded using the Jasco 815 CD spectropolarimeter at the Center for Macromolecular Interactions at Harvard Medical School. All peptides and vesicles were in 10 mM Na phosphate, pH 7.4, and 100 mM NaF buffer. Data were collected at ambient temperature with a scan speed of 50 nm/min, and 5 accumulations of each sample were averaged. The raw data were background subtracted for the presence or absence of vesicles and converted to mean residue ellipticity (MRE) and plotted using GraphPad Prism 9.0.
