# Erratum for Thorsen et al., “Highly Basic Clusters in the Herpes Simplex Virus 1 Nuclear Egress Complex Drive Membrane Budding by Inducing Lipid Ordering”

**DOI:** 10.1128/mbio.03673-21

**Published:** 2022-01-18

**Authors:** Michael K. Thorsen, Alex Lai, Michelle W. Lee, David P. Hoogerheide, Gerard C. L. Wong, Jack H. Freed, Ekaterina E. Heldwein

**Affiliations:** a Department of Molecular Biology and Microbiology, Graduate Program in Cellular, Molecular and Developmental Biology, Tufts University School of Medicine, Boston, Massachusetts, USA; b Department of Chemistry and Chemical Biology and National Biomedical Center for Advanced Electron Spin Resonance Technology, Cornell University, Ithaca, New York, USA; c Department of Bioengineering, Department of Chemistry and Biochemistry, California NanoSystems Institute, University of California, Los Angeles, Los Angeles, California, USA; d Center for Neutron Research, National Institute of Standards and Technology, Gaithersburg, Maryland, USA

## ERRATUM

Volume 12, no. 4, e01548-21, 2021, https://doi.org/10.1128/mbio.01548-21. Page 4: This erratum corrects [Fig fig1]Fig. 1F. The data for samples 185-His and 220d40-His were switched, and below is a new Fig. 1 to correct this mistake.

**Figure fig1:**
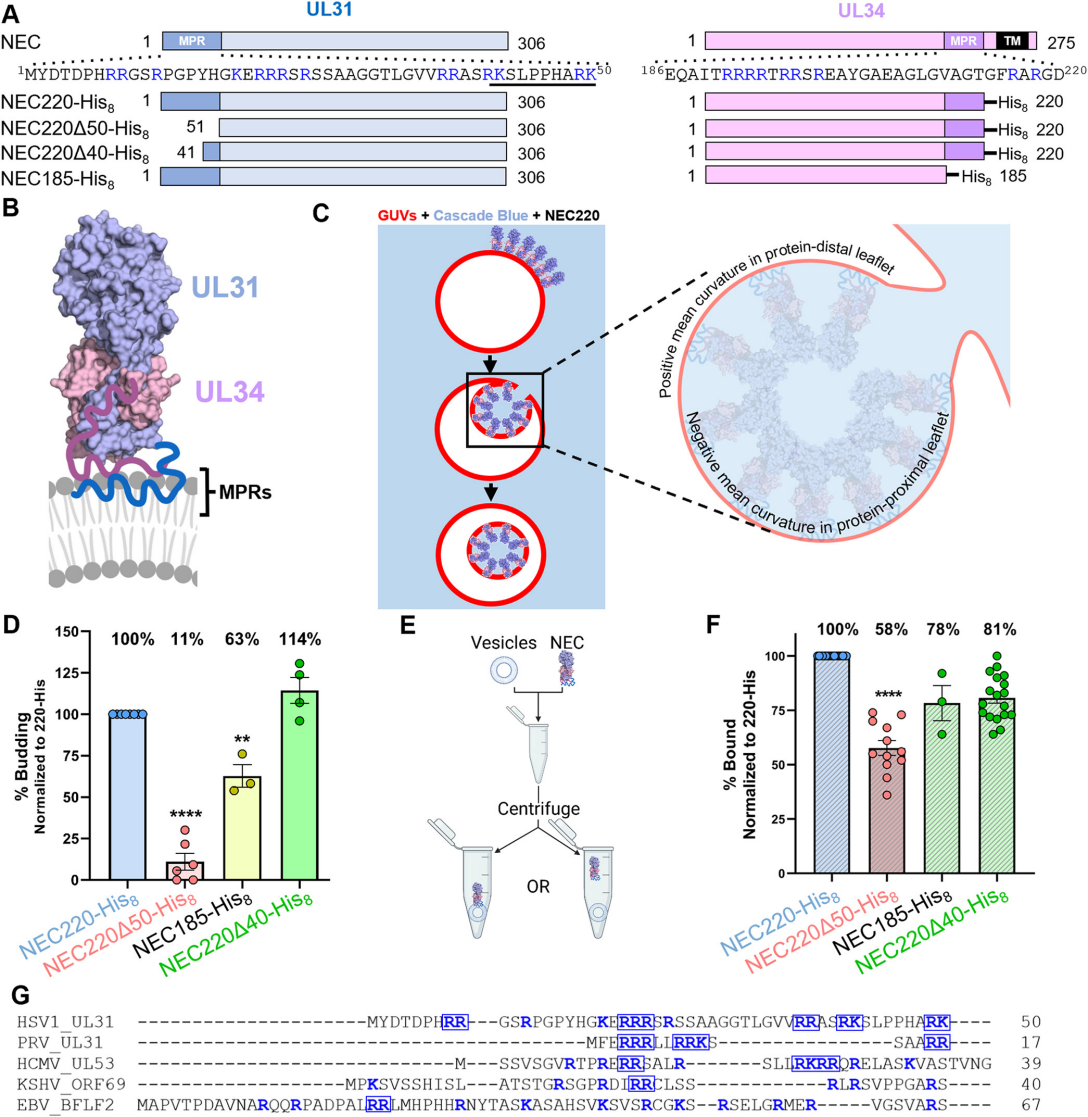
FIG 1

