# Supplementary material for: Highly Basic Clusters in the Herpes Simplex Virus 1 Nuclear Egress Complex Drive Membrane Budding by Inducing Lipid Ordering
Source: mBio. 2021 Aug 24;12(4):e01548-21. doi: 10.1128/mBio.01548-21 (PMC8406295; doi:10.1128/mBio.01548-21)
Supplement: TABLE S1 [file mbio.01548-21-st001.pdf]

**Parameter[a]**	**Value**	**68% CI[b]**
Sample broadening factor	1.98	[1.93, 2.03]
SiO_2 thickness (Å)	10.0	[6.1, 25.7]
Cr thickness (Å)	17.6	[8.2, 32.6]
Gold thickness (Å)	138.9	[133.9, 149.2]
SiO_2 SLD (10^{-6} Å^{-2})	3.6	[2.0, 4.3]
Cr SLD (10^{-6} Å^{-2})	3.3	[2.6, 3.6]
Au SLD (10^{-6} Å^{-2})	4.42	[4.38, 4.46]
Tether length (Å)	4.3	[3.0, 5.3]
Inner leaflet lipid length (Å)	15.3	[14.9, 17.5]
Outer leaflet lipid length (Å)	10.15	fixed [c]
Change in lipid thickness with protein (Å)[d]	0.49	[0.33, 0.66]
Fraction of exchangeable protons exchanged	0.921	[0.904, 0.960]
Number fraction of tether molecules in inner leaflet	0.84	[0.10, 0.93]
Ratio of b-mercaptoethanol to tether molecules	5.1	[1.3, 8.6]
Fractional completeness of bare bilayer	0.999	[0.988, 1.000]
Fractional completeness of bilayer with protein	0.985	[0.973, 0.989]
Number of protein molecules per outer leaflet lipid	0.022	[0.021, 0.023]
Distance of protein from headgroups (Å)	[e]	
Euler angle a		
Euler angle b		
MPR length	28.9	[26.1, 29.8]
MPR fractional position[f]	0.097	[0.020, 0.240]
Fraction of protein bound in H_2O spectrum	0.917	[0.870, 0.960]
SLD of D_2O solvent, bare bilayer (10^{-6} Å^{-2})	6.25	[6.17, 6.32]
SLD of H_2O solvent, bare bilayer (10^{-6} Å^{-2})	-0.564	[-0.565, -0.559]
SLD of D_2O solvent, bilayer + protein (10^{-6} Å^{-2})	6.24	[6.17, 6.31]
SLD of H_2O solvent, bilayer + protein (10^{-6} Å^{-2})	-0.345	[-0.390, -0.310]
Global roughness parameter (Å)	3.39	[3.04, 4.41]
Roughness of Cr/Au interface (Å)	14.1	[11.0, 18.2]
Roughness of bilayer (Å)	4.34	[3.79, 4.74]
Angle alignment correction (°)	0.000	[-0.003, 0.003]
Fractional beam intensity	0.959	[0.956, 0.961]
Background × 10^{-6}, D_2O solvent, bare bilayer	-0.089	[-0.230, 0.050]
Background × 10^{-6}, H_2O solvent, bare bilayer	2.90	[2.68, 3.18]
Background × 10^{-6}, D_2O solvent, bilayer + protein	-0.43	[-0.60, -0.23]
Background × 10^{-6}, H_2O solvent, bilayer + protein	2.69	[2.47, 2.97]

Supplementary Table S1. Parameters of the NR orientation model for 500 nM NEC220.

[a] For clarity, parameters for the Catmull-Rom freeform spline model are not shown.

[b] 68% confidence intervals are calculated from the stable solution to the MCMC optimization.

[c] Based on control measurement of bilayer alone.

[d] Defined in the model as the change in the inner lipid leaflet thickness.

[e] The two Euler angles a and b and the protein depth are highly correlated and are not reported as a value with standard errors. See Fig. 3E, G.

[f] The MPR was constrained to be near to the globular part of the protein. This parameter ranges from 1 (full extension of the MPR from the globular part of the protein) to 0 (full overlap of the MPR with the globular domains).
