# Supplementary material for: Highly Basic Clusters in the Herpes Simplex Virus 1 Nuclear Egress Complex Drive Membrane Budding by Inducing Lipid Ordering
Source: mBio. 2021 Aug 24;12(4):e01548-21. doi: 10.1128/mBio.01548-21 (PMC8406295; doi:10.1128/mBio.01548-21)
Supplement: TABLE S2 [file mbio.01548-21-st002.pdf]

Peptide	Sequence
UL31$^{(41-50)}$	^{41}RKSLPPHARK^{50}
UL31$^{(C40-50)}$	^{40}CRKSLPPHARK^{50}
UL31$^{(41-C51)}$	^{41}RKSLPPHARKC^{51}
UL31$^{scr(41-C51)}$	^{41}KSPKLHRARPC^{51}
UL31$^{(C40-50\ R41S/K42S)}$	^{40}CSSSLPPHARK^{50}
UL31$^{(22-42)}$	^{22}RRSRSSAAGGTLGVVRRASRK^{42}
UL31$^{(C21-42)}$	^{21}CRRSRSSAAGGTLGVVRRASRK^{42}
UL31$^{(22-C43)}$	^{22}RRSRSSAAGGTLGVVRRASRKC^{43}
UL31$^{scr(22-C43)}$	^{22}ARLGRSTSRGKSRAVRSVGRAC^{43}
UL31$^{(1-50)}$	^{1}MYDTDPHRRGSRPGPYHGKERRRSRSSAAGGTLGVVRRASRKSLPPHARK^{50}
UL31$^{(C1-50)}$	^{1}CYDTDPHRRGSRPGPYHGKERRRSRSSAAGGTLGVVRRASRKSLPPHARK^{50}
UL31$^{(1-C51)}$	^{1}MYDTDPHRRGSRPGPYHGKERRRSRSSAAGGTLGVVRRASRKSLPPHARKC^{51}
UL34$^{(174-194)}$	^{174}AGRTRRILCRAAEQAITRRRR^{194}
UL34$^{scr(174-194)}$	^{174}TRITRQCRAARGRERAARIRL^{194}

Supplementary Table S2. Sequences of UL31 and UL34 peptides used in CW-ESR experiments (Fig. 5)
