# Supplementary material for: Highly Basic Clusters in the Herpes Simplex Virus 1 Nuclear Egress Complex Drive Membrane Budding by Inducing Lipid Ordering
Source: mBio. 2021 Aug 24;12(4):e01548-21. doi: 10.1128/mBio.01548-21 (PMC8406295; doi:10.1128/mBio.01548-21)
Supplement: FIG S1 [file mbio.01548-21-sf001.pdf]

NEC peptide	Partition Ratio		
	N-terminal probe	C-terminal probe	Scrambled w/ C-terminal probe
**UL31$^{(41\text{-}50)}$**	0.818±0.033	0.875±0.023	0.75±0.086
**UL31$^{(41\text{-}50\ R41S/K42S)}$**	0.564±0.039	N/A	N/A
**UL31$^{(22\text{-}42)}$**	0.726±0.024	0.746±0.012	0.693±0.045
**UL34$^{(174\text{-}194)}$**	0.54±0.24 (Probe on C182)		0.52±0.032 (Probe on C180)

Supplementary Fig. S1. Membrane partition ratios of NEC MPR peptides. The partition ratio of the N- and C-terminal spin labeled UL31 and UL34 MPRs in the presence of POPC/POPS/POPA=3/1/1 SUV membranes. The partition ratios were calculated from the amount of spins in the supernatant and pellet of the double integral of the ESR signal. The averages and standard deviations (68% confidence intervals of the data) were calculated from three independent experiments.
