# Supplementary material for: Highly Basic Clusters in the Herpes Simplex Virus 1 Nuclear Egress Complex Drive Membrane Budding by Inducing Lipid Ordering
Source: mBio. 2021 Aug 24;12(4):e01548-21. doi: 10.1128/mBio.01548-21 (PMC8406295; doi:10.1128/mBio.01548-21)
Supplement: FIG S2 [file mbio.01548-21-sf002.pdf]

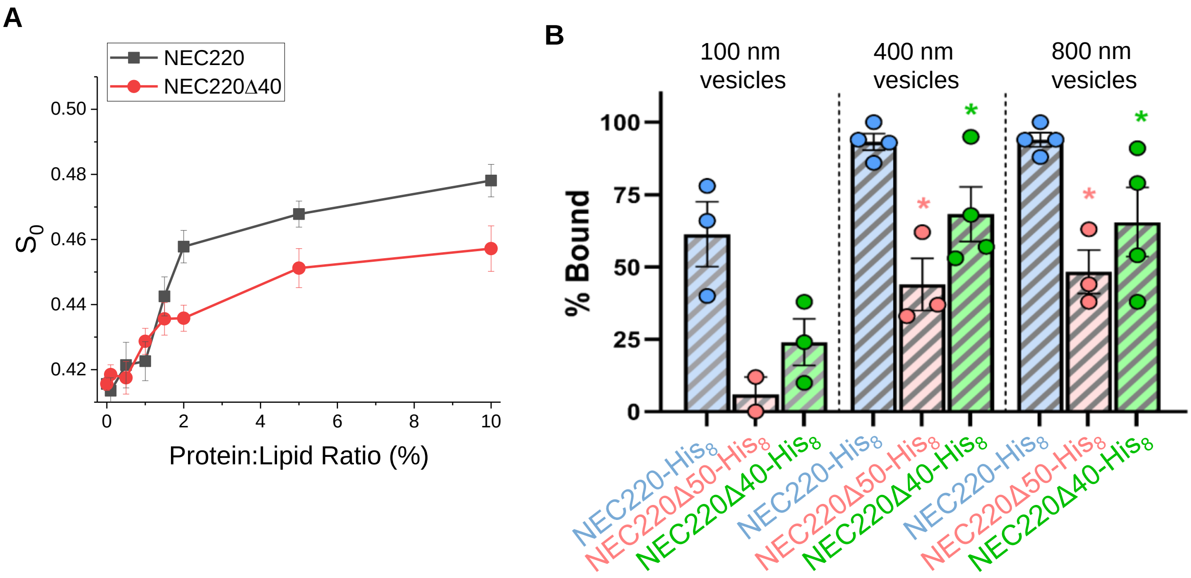

Supplementary Fig. S2. NEC220 and NEC220Δ40 induce lipid headgroup ordering and bind well to vesicles with less curvature. (A) Membrane ordering of DPPTC induced by NEC220 (black) and NEC220Δ40 (red). Error bars show the standard deviation (68% confidence interval of the data) from 3 independent experiments. (B) Purified NEC and vesicles of the indicated sizes containing 40% negatively charged lipids were co-floated by ultracentrifugation. Larger vesicles have smaller degrees of curvature. Each bar represents the amount of protein floating with vesicles in the top fraction minus background levels of floating protein in the absence of vesicles. Significance was calculated to binding of 100nm vesicles for each respective construct using an unpaired Student's t-test with Welch's correction ($p<0.05$=*). Error bars represent the standard error of the mean (68% confidence interval of the mean) for at least two individual experiments.
