# Supplementary material for: Highly Basic Clusters in the Herpes Simplex Virus 1 Nuclear Egress Complex Drive Membrane Budding by Inducing Lipid Ordering
Source: mBio. 2021 Aug 24;12(4):e01548-21. doi: 10.1128/mBio.01548-21 (PMC8406295; doi:10.1128/mBio.01548-21)
Supplement: FIG S3 [file mbio.01548-21-sf003.pdf]

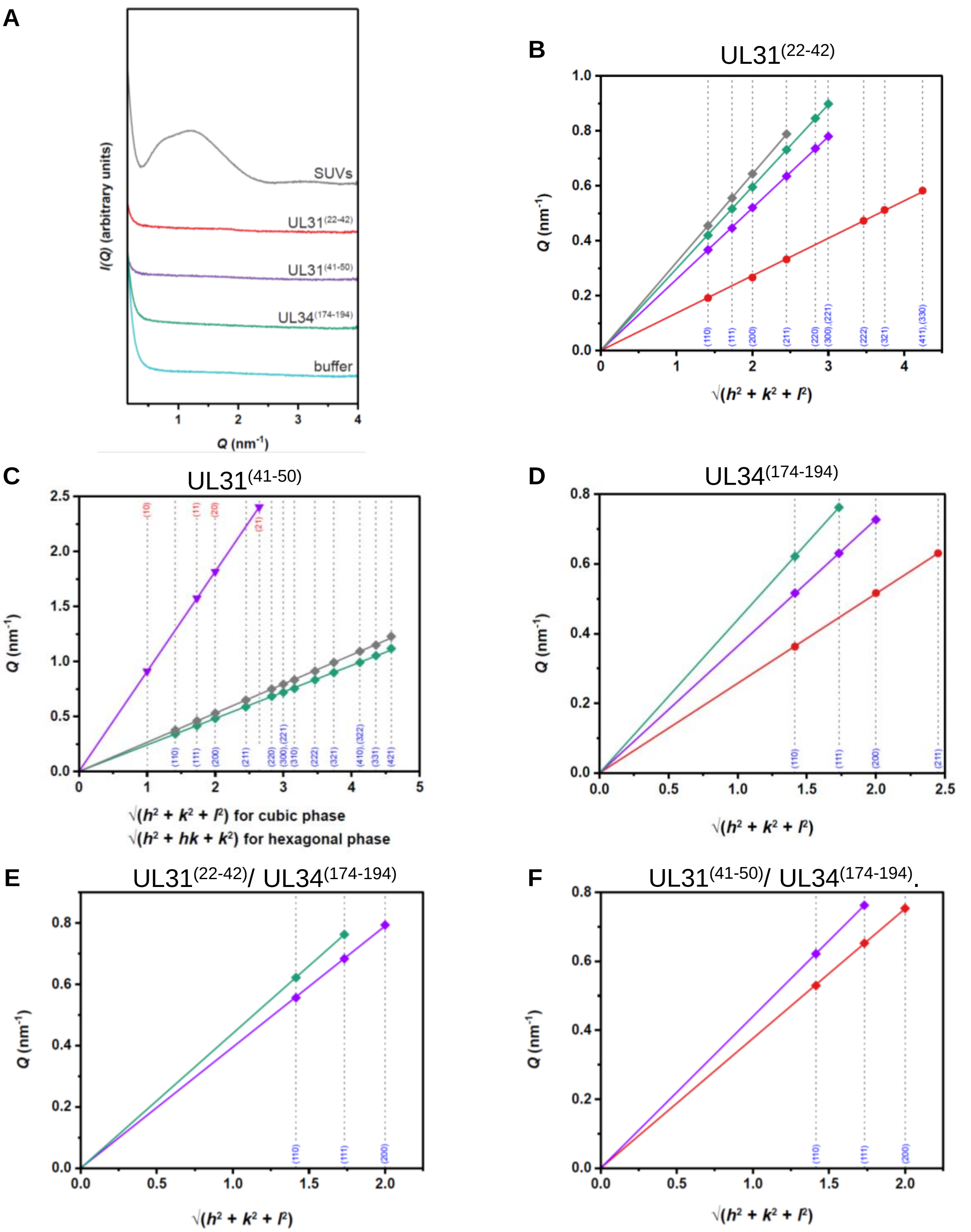

Supplementary Fig. S3. SAXS spectra for control and indexing of the identified liquid-crystalline lipid phases. (A) Spectra for SUVs in buffer, individual peptides in buffer, and buffer-only controls. For improved visualization, spectra have been manually offset in the vertical direction by scaling each trace by a multiplicative factor. Linear regressions of indexed reflections for phases induced by **(B)** UL31$^{(22\text{-}42)}$, **(C)** UL31$^{(41\text{-}50)}$, **(D)** UL34$^{(174\text{-}194)}$, **(E)** UL31$^{(22\text{-}42)}$/ UL34$^{(174\text{-}194)}$, and **(F)** UL31$^{(41\text{-}50)}$/ UL34$^{(174\text{-}194)}$. **(B–F)** Vertical dotted reference lines denote indexed reflections. Each plotted point corresponds to an indexed peak, with its associated lattice indicated by the marker symbol (circle = *Im3m* cubic, diamond = *Pn3m* cubic, triangle = hexagonal). Colors of each regression correspond to the same ch P/L values in Fig. 7. Calculated lattice parameters and NGC values for the identified phases are provided in Supplementary Fig. S4.
