# Supplementary material for: Highly Basic Clusters in the Herpes Simplex Virus 1 Nuclear Egress Complex Drive Membrane Budding by Inducing Lipid Ordering
Source: mBio. 2021 Aug 24;12(4):e01548-21. doi: 10.1128/mBio.01548-21 (PMC8406295; doi:10.1128/mBio.01548-21)
Supplement: FIG S4 [file mbio.01548-21-sf004.pdf]

UL31(22-42)

Charge P/L<br>Molar P/L	1/4<br>1/140	1/2<br>1/70	1/1<br>1/35	3/2<br>3/70	2/1<br>2/35
Cubic	------	*Im3m*: 46.08 nm<br>|<*K*>| = 5.05e-3 nm^{-2}	*Pn3m*: 24.20 nm<br>|<*K*>| = 1.12e-2 nm^{-2}	*Pn3m*: 21.04 nm<br>|<*K*>| = 1.48e-2 nm^{-2}	*Pn3m*: 19.54 nm<br>|<*K*>| = 1.72e-2 nm^{-2}
Hexagonal	------	------	------	------	------
Lamellar	------	7.17 nm	6.43 nm	6.26 nm	6.20 nm

UL31(41-50)

Charge P/L<br>Molar P/L	1/4<br>1/80	1/2<br>1/40	1/1<br>1/20	3/2<br>3/40	2/1<br>1/10
Cubic	------	------	------	*Pn3m*: 26.09 nm<br>|<*K*>| = 9.62e-3 nm^{-2}	*Pn3m*: 23.73 nm<br>|<*K*>| = 1.16e-2 nm^{-2}
Hexagonal	------	------	7.98 nm	------	------
Lamellar	------	------	------	------	------

UL34(174-194)

Charge P/L<br>Molar P/L	1/4<br>1/140	1/2<br>1/70	1/1<br>1/35	3/2<br>3/70	2/1<br>2/35
Cubic	------	*Im3m*: 24.41 nm<br>|<*K*>| = 1.80e-2 nm^{-2}	*Pn3m*: 17.26 nm<br>|<*K*>| = 2.20e-2 nm^{-2}	*Pn3m*: 14.28 nm<br>|<*K*>| = 3.21e-2 nm^{-2}	------
Hexagonal	------	------	------	------	------
Lamellar	7.92 nm	6.86 nm	6.07 nm	6.00 nm	6.02 nm

UL31(22-42)/ UL34(174-194)

UL31(22-42)<br>UL34(174-194)	Molar P/L = 1/35<br>Molar P/L = 1/35	Molar P/L = 3/70<br>Molar P/L = 3/70
Cubic	*Pn3m*: 15.91 nm<br>|<*K*>| = 2.59e-2 nm^{-2}	*Pn3m*: 14.28 nm<br>|<*K*>| = 3.21e-2 nm^{-2}
Hexagonal	------	------
Lamellar	6.02 nm	6.00 nm

UL31(41-50)/ UL34(174-194)

UL31(41-50)<br>UL34(174-194)	Molar P/L = 1/40<br>Molar P/L = 1/35	Molar P/L = 1/20<br>Molar P/L = 3/70
Cubic	*Pn3m*: 16.70 nm<br>|<*K*>| = 2.35e-2 nm^{-2}	*Pn3m*: 14.28 nm<br>|<*K*>| = 3.21e-2 nm^{-2}
Hexagonal	------	------
Lamellar	6.02 nm	5.97 nm

Supplementary Fig. S4. Phase diagram with tabulated lattice parameters and negative Gaussian curvature values for each SAXS condition. Cells containing dashed lines indicate that the phase was either not present or could not be indexed due to insufficient number of reflections.
