# Supplementary material for: Highly Basic Clusters in the Herpes Simplex Virus 1 Nuclear Egress Complex Drive Membrane Budding by Inducing Lipid Ordering
Source: mBio. 2021 Aug 24;12(4):e01548-21. doi: 10.1128/mBio.01548-21 (PMC8406295; doi:10.1128/mBio.01548-21)
Supplement: FIG S5 [file mbio.01548-21-sf005.pdf]

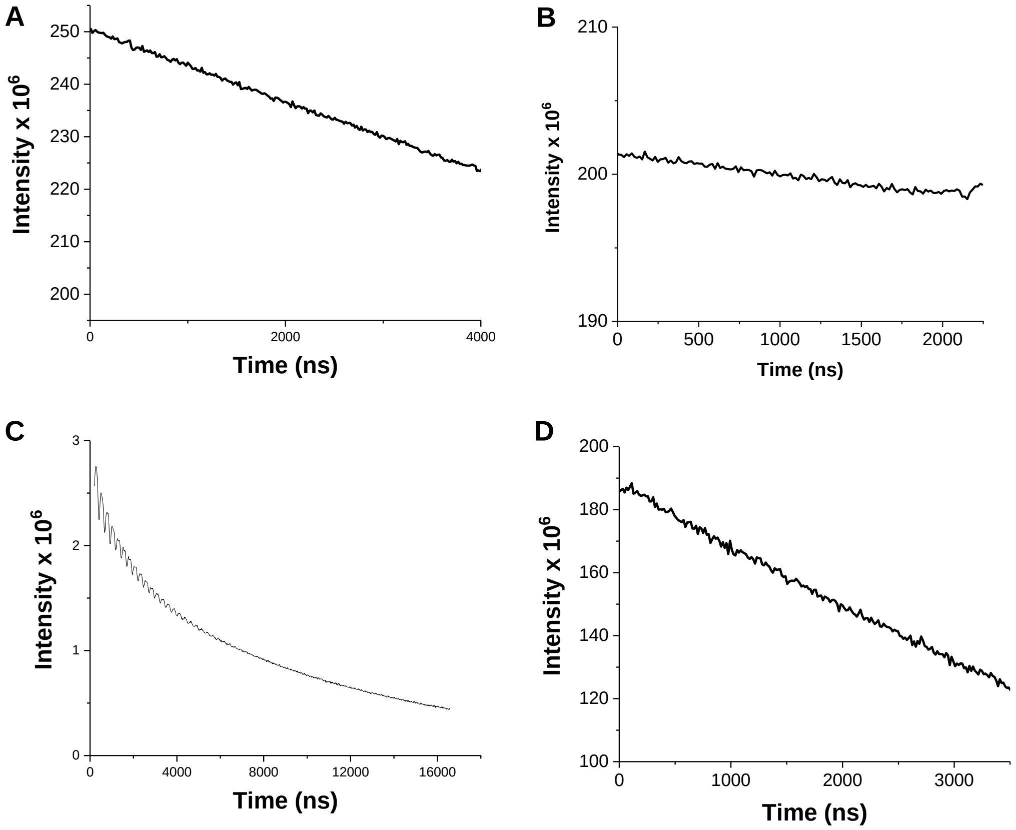

Supplementary Fig. S5. DEER and spin echo signals. (A) DEER signal for UL31$^{(C1\text{-}50)}$ in membranes. (B) DEER signal for UL34$^{(174\text{-}194)}$ in membranes (C) Spin echo signal for UL31$^{(C1\text{-}50)}$ in membranes. (D) DEER signal for UL31$^{(C1\text{-}50)}$/UL34$^{(174\text{-}194)}$ in solution.
