# Supplementary material for: Highly Basic Clusters in the Herpes Simplex Virus 1 Nuclear Egress Complex Drive Membrane Budding by Inducing Lipid Ordering
Source: mBio. 2021 Aug 24;12(4):e01548-21. doi: 10.1128/mBio.01548-21 (PMC8406295; doi:10.1128/mBio.01548-21)
Supplement: FIG S6 [file mbio.01548-21-sf006.pdf]

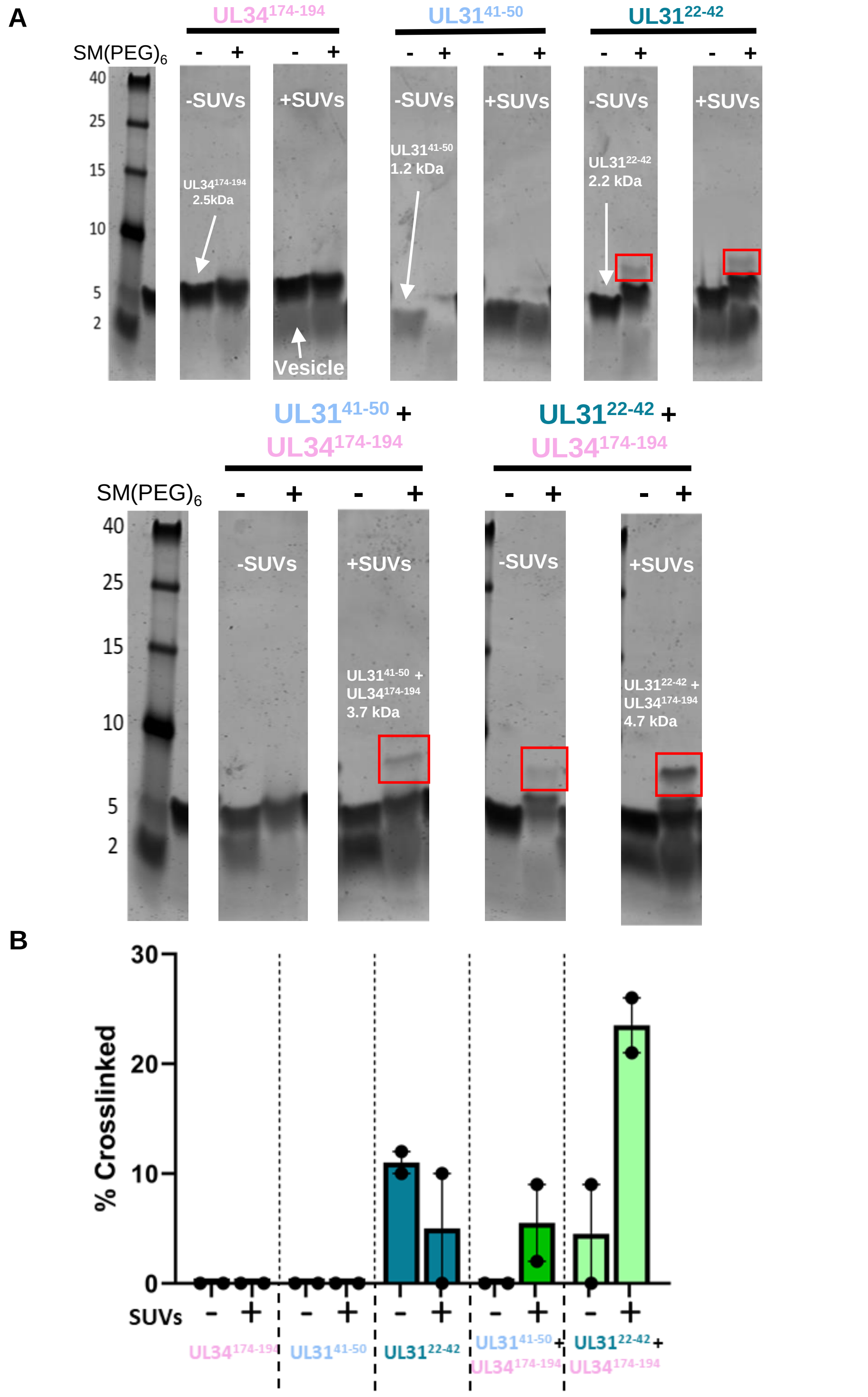

Supplementary Fig. S6. Crosslinking of UL31 and UL34 MPR peptides. (A) Individual and combination of peptides were crosslinked in the absence or presence of 3/1/1=POPC/POPS/POPA SUVs in two individual experiments. SM(PEG)$_6$ was added at 50-fold molar excess. Samples were analyzed by 16% Tricine-SDS-PAGE and Coomassie staining. Samples were run on gels grouped by presence or absence of SUVs. Images depict representative gels from one experiment where lanes are cropped to re-group based on peptides used rather than presence or absence of vesicles. Predicted molecular masses of individual peptides and 1:1 complexes are indicated. Red boxes denote crosslinked UL31-UL34 peptides. (B) Quantification of two individual experiments. Each bar represents the amount of peptide crosslinked. Error bars represent the standard error of the mean (68% confidence interval of the mean) for two individual experiments.
