# Supplementary material for: Highly Basic Clusters in the Herpes Simplex Virus 1 Nuclear Egress Complex Drive Membrane Budding by Inducing Lipid Ordering
Source: mBio. 2021 Aug 24;12(4):e01548-21. doi: 10.1128/mBio.01548-21 (PMC8406295; doi:10.1128/mBio.01548-21)
Supplement: FIG S7 [file mbio.01548-21-sf007.pdf]

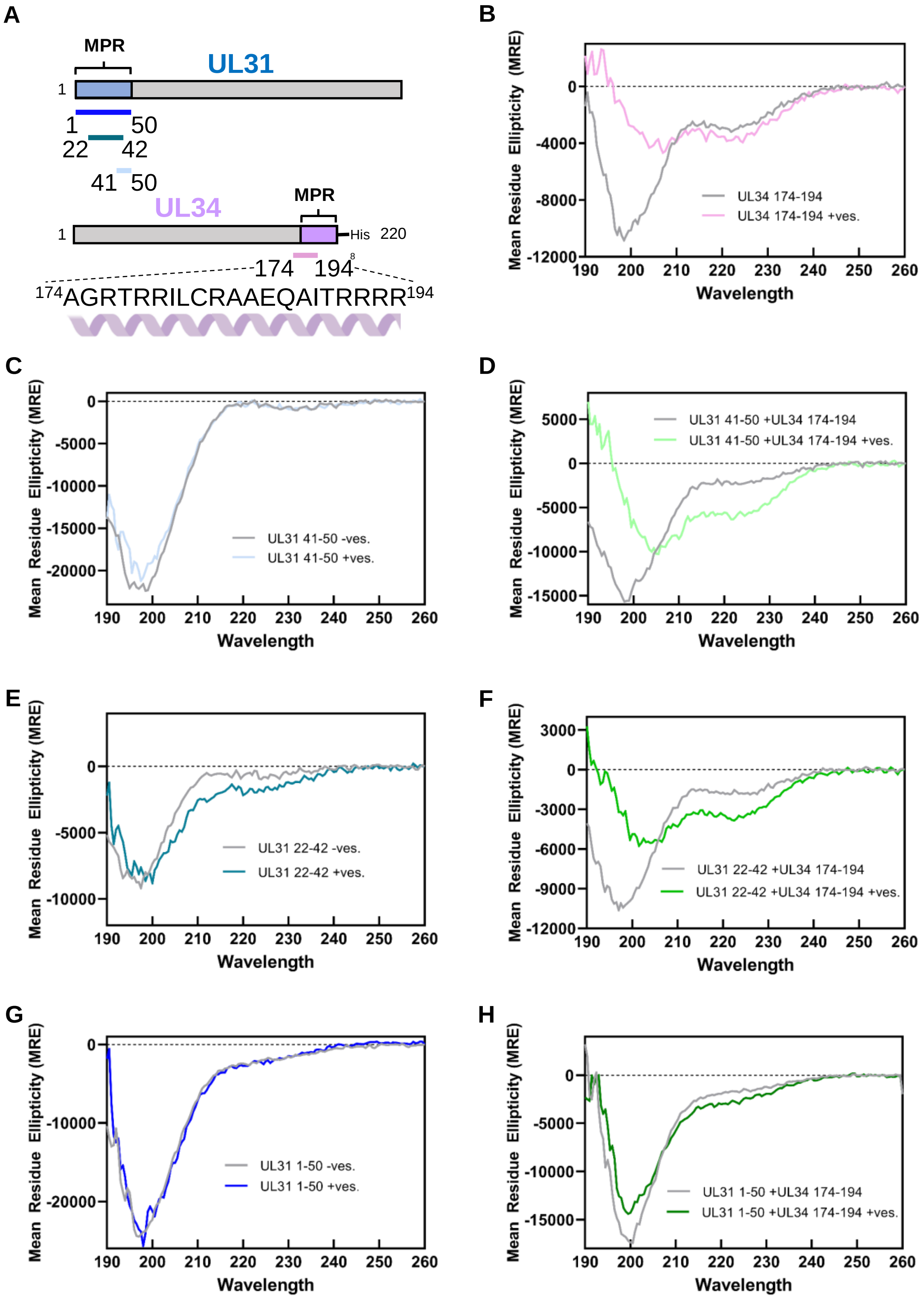

Supplementary Fig. S7. Circular dichroism of UL31 and UL34 MPR peptides. (A) Schematic illustrating peptide location in NEC sequence. UL34 174-194 sequence and predicted secondary structure shown underneath schematic. No part of UL31 MPR is predicted to form secondary structure. (B-H) Overlays of the far-UV CD spectra in the presence and absence of POPC/POPS/POPA 3:1:1 SUVs. Averages of five replicates are reported.
