# Supplementary material for: Highly Basic Clusters in the Herpes Simplex Virus 1 Nuclear Egress Complex Drive Membrane Budding by Inducing Lipid Ordering
Source: mBio. 2021 Aug 24;12(4):e01548-21. doi: 10.1128/mBio.01548-21 (PMC8406295; doi:10.1128/mBio.01548-21)
Supplement: TABLE S3 [file mbio.01548-21-st003.pdf]

Plasmid	Construct Info	Forward Primer	Reverse Primer
pJB02	HSV1 UL34 1-220	5'-aaaaaagtcgacctatggcgggactgggcaag-3'<br>SalI	5'-aaaaaagcggccgccttcagtcccccctggccc-3'<br>NotI
pJB57	HSV1 UL34 1-220-His_8	5'-aaaaaagtcgacctatggcgggactgggcaag-3'<br>SalI	5'-aaaaaagcggccgccttcagtcccccctggccc-3'<br>NotI
pJB84	HSV UL34 1-185-His_8	5'-aaaaaagtcgacctatggcgggactgggcaag-3'<br>SalI	5'-aaaaaagcggccgcctggcggcgcggcaca-3'<br>NotI
pKH90	HSV1 UL31 f.l.	5'-agcaggatcctatgacaccgaccccccat-3'<br>BamHI	5'-aaatgcggccgcttacggcggaggaaactc-3'<br>NotI
pKH86	HSV1 UL31 51-306	5'-agcaggatcccaggagctgtgtttacac-3'<br>BamHI	5'-aaatgcggccgcttacggcggaggaaactc-3'<br>NotI
pJB41	HSV1 UL31 41-306	5'-aaaaaaggatcccggaagagcctgccg-3'<br>BamHI	5'-aaatgcggccgcttacggcggaggaaactc-3'<br>NotI
pMT02	HSV1 UL31-Δ40 R49S/K50S	5'-**agc**caggagctgtgtttacacgagcgc-3'	5'-**gct**ggcgtgaggcggcaggctctt-3'
pMT08	HSV1 UL31-Δ40 R41S/K42S/S43R/L44K/R49S/K50S	5'-**cgtaaa**ccgcctcacgccagc-3'	5'-**gctgct**ggaggatccgggccc-3'
pMT09	HSV1 UL31-Δ40 R41S/K42S/P45R/P46K/R49S/K50S	5'-**gctgct**ggaggatccgggccc-3'	5'-agcctg**cgtaaa**cacgccagcag-3'
pMT10	HSV1 UL31-Δ40 R41S/K42S/H47R/A48K/R49S/K50S	5'-agcctgccgcct**cgtaaa**agc-3'	5'-**gctgct**ggaggatccgggccc-3'
pMT03	HSV1 UL31-Δ40 R41S/K42S	5'-aaaaaaggatcctcc**tcttct**agcctgccgcc-3'<br>BamHI	5'- aaatgcggccgcttacggcggaggaaactc-3'<br>NotI
pMT01	HSV1 UL31-Δ40 R41S/K42S/R49S/K50S	5'-cctcacgcc**tcttct**caggagctgtgttt-3'	5'-cggcaggct**agaaga**ggaggatc-3'
pMT17	HSV1 UL31-Δ40 L44A	5'-agc**gcg**ccgcctcacgccc-3'	5'-cttccgggatccgggcc-3'
pMT11	HSV1 UL31-Δ40 P45A/P46A	5'-agcctg**gcggcg**cacgcccgcaaa-3'	5'-cttccgggatccgggcc-3'
pMT18	HSV1 UL31-Δ50 RKRK	5'-agctgtgtctgcacgagcgc-3'	5'-cctgtttgcg**tttacg**ggatccggg-3'
pMT35	HSV1 UL31-Δ40 Scr 41-50	5'-**aaaagcccgaaactgcatcgtgcgcgtccg**caggagctgtgtttacacgagcg-3'	5'-ggatccgggcccctggaaca-3'
pMT04	HSV1 UL31-Δ40 S43E	5'-**gaa**ctgccgcctcacgc-3'	5'-cttccgggatccgggcc-3'
pJB60	HSV1 UL31 2-306 S11E/S24E/S26E/S27E/S40E/S43E	5'-cccccatcgccgcggc**gaa**cggcccgggccctatcac-3'	5'-gtgatagggcccgggccg**ttc**gccgcggcgatggggg-3';
	Round 2	5'-gagcgccggcgg**gaa**cgc**gaagaa**gcggccggcggg-3'	5'-cccgccggccgc**ttcttc**gcg**ttc**ccgccggcgctc-3'
	Round 3	5'-cgtggtgcgtcgggcc**gaa**cggaag**gaa**ctgccgcctcacgccc-3'	5'-gggcgtgaggcggcag**ttc**cttccg**ttc**ggcccgacgcaccacg-3'
pMT16	HSV-1 UL31 S11E/S24E/S43E	5'-gcaaggagcgccggcgg**gaa**cgctc-3'	5'-cgtgatagggcccgggccg**ttc**gcc-3'
	Round 2	5'-gggccctatcacggcaaggagc-3'	5'-gggccg**ttc**gccgcggcgatg-3'
pMT19	HSV-1 UL31 1-50	5'-aaaaaaggatcctgctcctatgacaccgacccc-3'<br>BamHI	5'-aaaaaagcggccgcttattatttgcgggcgtgaggcg-3'<br>NotI
pMT29	HSV-1 UL31 C1-50	5'-ctgttccaggggcccggatcc**tgc**tatg-3'	5'-cata**gca**ggatccgggcccctggaacag-3'
	Round 2	5'-aggggcccggatcc**tgc**tatgacaccgacc-3'	5'-ggtcggtgtcata**gca**ggatccgggcccct-3'
	Round 3	5'-cagagatttcaatgcttgaaggagcggt-3'	5'-accgctccttcaagcattgaaatctctg-3'
pMT33	HSV-1 UL31 1-C51	5'-cacgcccgcaaat**tgc**taataagcggc-3'	5'-gccgcttatta**gca**tttgcgggcgtg-3'

Supplementary Table S3. List of primers used for cloning described in Materials and Methods. All primers are listed in the 5'-3' direction. Mutations are bolded, restriction digest sites are underlined and listed underneath applicable primers.
